# Clinical whole-genome sequencing from routine formalin-fixed, paraffin-embedded specimens: pilot study for the 100,000 Genomes Project

**DOI:** 10.1038/gim.2017.241

**Published:** 2018-02-01

**Authors:** Pauline Robbe, Niko Popitsch, Samantha J.L. Knight, Pavlos Antoniou, Jennifer Becq, Miao He, Alexander Kanapin, Anastasia Samsonova, Dimitrios V Vavoulis, Mark T. Ross, Zoya Kingsbury, Maite Cabes, Sara D.C. Ramos, Suzanne Page, Helene Dreau, Kate Ridout, Louise J Jones, Alice Tuff-Lacey, Shirley Henderson, Joanne Mason, Francesca M Buffa, Clare Verrill, David Maldonado-Perez, Ioannis Roxanis, Elena Collantes, Lisa Browning, Sunanda Dhar, Stephen Damato, Susan Davies, Mark Caulfield, David R. Bentley, Jenny C. Taylor, Clare Turnbull, Anna Schuh

**Affiliations:** 1 Oxford Molecular Diagnostics Centre, Radcliffe Department of Medicine, University of Oxford, Oxford, UK; 2 Wellcome Trust Centre of Human Genetics, Old Road Campus Research Building, University of Oxford, Oxford, UK; 3 Illumina Cambridge Ltd., Chesterford Research Park, Saffron Walden, UK; 4 Department of Oncology, University of Oxford, Oxford, UK; 5 Oxford Molecular Diagnostics Centre, John Radcliffe Hospital, Oxford University Hospitals NHS Trust, Oxford, UK; 6 Genomics England, William Harvey Research Institute, Queen Mary University of London, London, UK; 7 Computational Biology and Integrative Genomics, Department of Oncology, University of Oxford, Oxford, UK; 8 Nuffield Department of Surgical Sciences, University of Oxford, John Radcliffe Hospital, Oxford, UK; 9 Department of Cellular Pathology, Oxford University Hospital Foundation Trust, Oxford, UK; 10 NIHR Biomedical Research Centre at Barts Health NHS Trust, London, UK; 11 NIHR Comprehensive Biomedical Research Centre, Oxford, UK; 12 Division of Genetics and Epidemiology, Institute of Cancer Research, London, UK; 13 Oxford Molecular Diagnostics Centre, Department of Oncology, University of Oxford, Oxford, UK

**Keywords:** clinical variant reporting, copy-number alteration, formalin-fixed, paraffin-embedded (FFPE), somatic variants, whole-genome sequencing

## Abstract

**Purpose:**

Fresh-frozen (FF) tissue is the optimal source of DNA for whole-genome sequencing (WGS) of cancer patients. However, it is not always available, limiting the widespread application of WGS in clinical practice. We explored the viability of using formalin-fixed, paraffin-embedded (FFPE) tissues, available routinely for cancer patients, as a source of DNA for clinical WGS.

**Methods:**

We conducted a prospective study using DNAs from matched FF, FFPE, and peripheral blood germ-line specimens collected from 52 cancer patients (156 samples) following routine diagnostic protocols. We compared somatic variants detected in FFPE and matching FF samples.

**Results:**

We found the single-nucleotide variant agreement reached 71% across the genome and somatic copy-number alterations (CNAs) detection from FFPE samples was suboptimal (0.44 median correlation with FF) due to nonuniform coverage. CNA detection was improved significantly with lower reverse crosslinking temperature in FFPE DNA extraction (80 °C or 65 °C depending on the methods). Our final data showed somatic variant detection from FFPE for clinical decision making is possible. We detected 98% of clinically actionable variants (including 30/31 CNAs).

**Conclusion:**

We present the first prospective WGS study of cancer patients using FFPE specimens collected in a routine clinical environment proving WGS can be applied in the clinic.

## Introduction

With the progress in analytical capability and cost reduction, it is widely accepted that whole-genome sequencing (WGS) presents advantages over targeted platforms.^1^,^2^ WGS, a single test, is particularly valuable for investigating all variant types, including single-nucleotide variants (SNVs), small insertions/deletions (indels), and structural variants such as copy-number alterations (CNAs). Indeed, the conventional multi-modality testing currently employed in routine diagnostics can rapidly exhaust the low amount of material available from tumor specimens.^3^,^4^ Although several targeted sequencing methods also allow the detection of all classes of mutations, WGS presents an additional advantage of unbiased sequencing.^5^–^7^ The comprehensive nature of WGS also removes the need to redesign and validate additional tests.

Previous studies have demonstrated the feasibility of WGS for cancer patients in the clinic^8^ but focused on using high-quality nucleic acids extracted from fresh-frozen tissue (FF) specimens collected within a research infrastructure.^9^ However, FF specimens are not routinely collected because formalin-fixed, paraffin-embedded (FFPE) material is the specimen of choice for histopathological diagnosis.^10^ DNA extracted from FFPE specimens presents degradation due to specimen processing^11^ such as nucleic acid fragmentation, DNA crosslinks, abasic sites leading to localized DNA denaturation and strand breaks, and deamination leading to C > T mutation artifacts,^12^ which impede downstream sequencing analysis.

Several studies have compared sequencing data obtained from FF and FFPE specimens and have shown that FFPE samples can be interrogated using targeted sequencing, including whole-exome approaches.^13^–^20^ However, there is a paucity of data evaluating WGS data.^17^,^21^,^22^ Furthermore, most of these studies did not use a matched normal sample as germ-line (GL) control and therefore somatic variants detection has not been investigated rigorously. Only two studies performing whole-exome sequencing considered somatic SNVs (using GL sample data) in exonic regions in four cancer samples.^18^,^19^

Previous studies have disagreed as to whether CNA calls between FF and FFPE were comparable^16^,^22^ or poorer in FFPE samples.^13^ In the first two studies, low-depth WGS or whole-exome sequencing without matching GL samples were used and in the third study whole-exome sequencing of one patient (FF, FFPE, and matched GL samples) was reported.

Therefore, there is a lack of knowledge regarding somatic mutations detection performance using WGS from FFPE specimens (in particular structural variants and variants in noncoding regions).

In the present study, we addressed key technical questions relating to WGS analysis of DNA from FFPE-derived cancer. We present the largest study to date evaluating WGS data sets obtained from 156 genomes from 52 matched FF tumor, FFPE tumor, and peripheral blood GL samples routinely collected as part of the diagnostic process. We detail comprehensively the differences observed between FF and FFPE sequence data and propose a method to optimize the quality of FFPE-derived WGS data that will allow the acquisition of genome-wide data for all patients with cancer, including those for whom only FFPE material is available.

## Materials and Methods

### Sample collection and processing

One hundred eighty-four consecutive patients with cancer undergoing surgical resection with curative intent were recruited with written informed consent for research use at the Oxford University Hospitals Foundation Trust’s Genomics Medicine Centre. Studies, conducted in accordance with the Declaration of Helsinki, were approved by all relevant institutional ethical committees and regulatory review bodies. The tissue samples collected from adjacent region of tissue blocks were prepared as both FF and FFPE samples following the usual protocol in National Health Service diagnostic laboratories. A peripheral blood sample (2 ml) was collected for each patient (Supplementary Methods online). Nucleic acids extraction and quality control are detailed in the Supplementary Methods.

### Whole-genome sequencing

TruSeq DNA PCR-Free libraries were prepared from blood and FF tissues using 1 μg of input DNA according to the manufacturer’s instructions (Illumina, San Diego, CA). FFPE specimens were prepared using the Illumina FFPE-extracted genomic DNA sample preparation and TruSeq Nano DNA Library Prep (Illumina, San Diego, CA, USA), according to the manufacturer’s instructions (Supplementary Methods). Sequencing was performed on a HiSeq2500 (Illumina, San Diego, CA, USA) to an average depth of coverage of 70 × for tumor samples and 30 × for GL samples. Alignment is detailed in the Supplementary Methods.

### Somatic SNV, indel, and CNA calling

Somatic SNV detection was performed with Mutect v1.1.4,^23^ Shimmer v0.1.1,^24^ and Strelka 2.0.14.^25^ Indel detection was performed using Shimmer and Strelka. The combination method called variants considered “high confidence” as they were identified by all variant callers according to VCF Intersect. (Supplementary Methods). Variants were validated using the AmpliSeq Cancer Hotspot Panel (Supplementary Methods).

Log_2_R was calculated from tumor-normal pair data using BIC-seq v1.2.1.^26^ Somatic CNAs were called and manually curated with Nexus Discovery Edition 7.5 (BioDiscovery, El Segundo, CA, USA) (Supplementary Methods).

### FFPE DNA extraction optimization

Several reverse crosslinking temperatures (90 °C, 80 °C, 65 °C, and 56 °C), incubation times (1 and 3 h), and buffer addition (saline sodium citrate) were studied in 33 samples from 5 patients (Supplementary Table S1) using two DNA extraction kits (Supplementary Table S2). To ensure homogeneity of the different sample conditions extracted, all proteinase K digested aliquots from the same patients were pooled, homogenized, and split before the reverse crosslinking step (Supplementary Methods).

### Clinical reporting

Clinical reports were generated for five renal cases from FF and independently from matching FFPE data. SNVs and indels from Strelka 2.0.14 (ref. 25) were annotated using VEP GRCh37 release 85 (ref. 27) and only variants in the COSMIC cancer gene census^28^ (referencing 600 genes), and genes of the renal cell carcinoma and PI3K-Akt signaling KEGG pathways were kept for further analysis.^29^ CNA calls were manually curated using Nexus Discovery Edition 7.5 and annotated against the different gene list of interest (detailed for SNVs and indels). Alterations were divided into tiers to determine clinically actionable ones (druggable/predictive/prognostic and/or with diagnostic/classification implications^30^) (Supplementary Methods).

## Results

### A significant number of samples lost to the study due to poor quality

A GL sample from peripheral blood and two tumor specimens prepared as FF and FFPE samples were required for each patient. Of 184 patients, 87 (48%) were excluded due to lack of a suitable FF sample and 30 of the remaining patients were excluded because of the poor quality of FFPE DNAs or libraries. Another 15 patients were excluded at various other quality control (QC) steps. Trio sets were available for 52 cancer patients (10 breast, 12 colorectal, 7 endometrial, 4 prostatic, 14 renal, and 5 thoracic tissues) (Supplementary Figure S1A,B, Supplementary Table S3).

### FFPE DNA quality control revealed short and denatured DNA

The quantity and quality of the 52 matching FF and FFPE DNAs were studied (Supplementary Table S4, Wilcoxon signed rank test). The total nucleic acid yield assessed using Nanodrop (Thermo Fisher Scientific, MA), measuring all nucleic acids, was lower for FFPE than FF DNAs, without reaching statistical significance (*p* = 0.0781). The same metric measured by Qubit dsDNA Broad Range Assay kit (Thermo Fisher Scientific), assessing only double-stranded DNA, showed a significantly lower DNA quantity for FFPE samples (*p* = 1.41e^− 06^). These results supported the assertion that FFPE samples yielded similar amounts of total nucleic acids, but lowered double-stranded DNA quantity due to denaturation. The A260/A280 ratio, a marker of nucleic acid purity, was similar between FFPE and FF samples with mean values of 1.89 and 1.88, respectively. However, when subjected to gel electrophoresis the FFPE DNAs revealed nucleic acid smears in the range <0.5–40 kb, indicating DNA fragmentation, whereas the matching FF samples presented a distinctive band > 40 kb (Supplementary Figure S2A,B).

### FFPE DNA presented shorter fragments and sequencing data revealed nonuniform coverage

**Figure 1:**
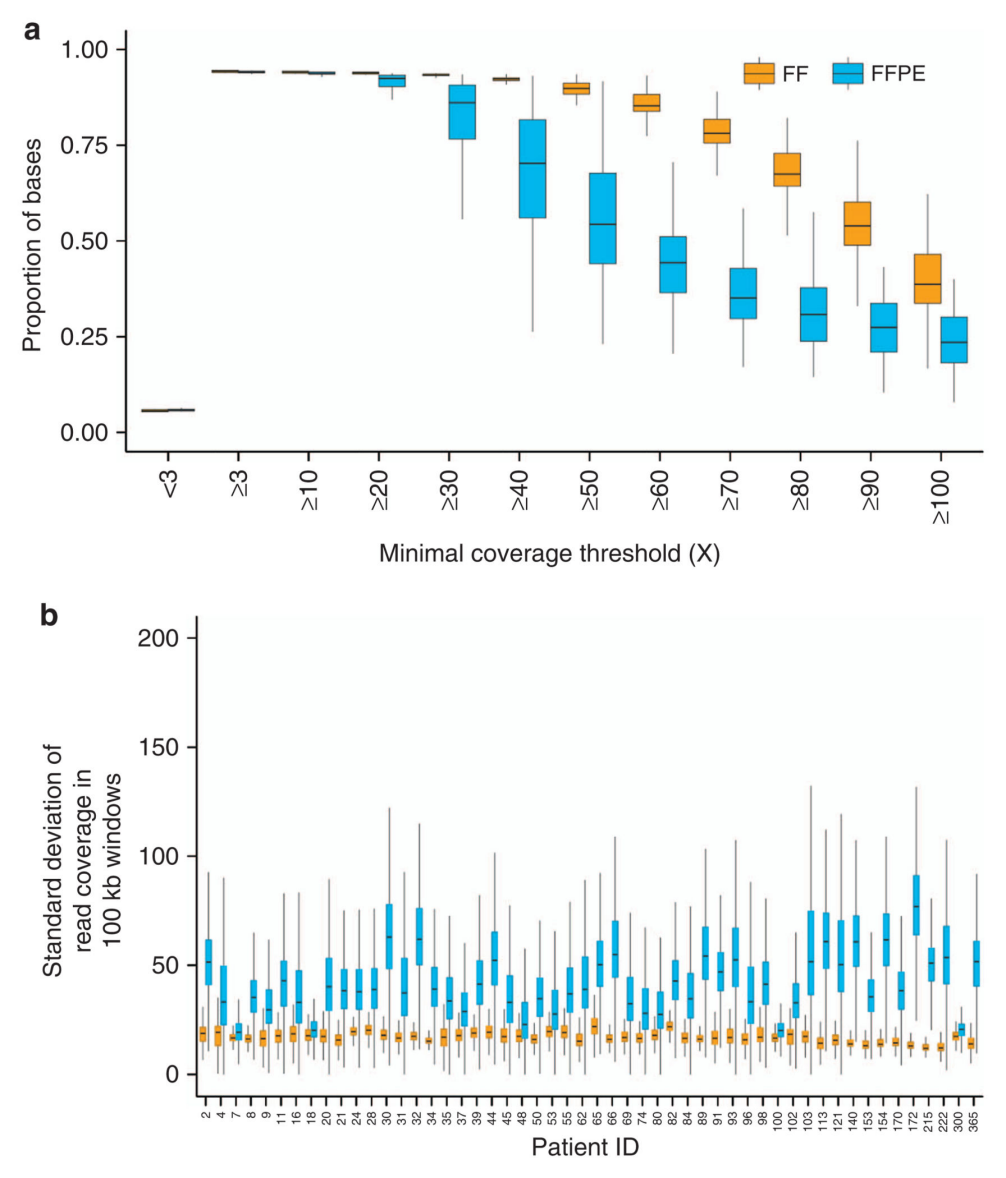
Sequencing depth statistics. (**a**) Distribution of sequencing depth for different minimum coverage thresholds for 52 FF and 52 matching FFPE samples. The sequencing coverage aim for tumor samples was 70 ×. (**b**) Distribution of standard deviations of sequencing coverage in 100-kb genomic windows for FF and matching FFPE samples. Higher values denote poor coverage uniformity, which directly impacts variant detection. FF, fresh-frozen sample; FFPE, formalin-fixed, paraffin-embedded sample.

Alignment metrics reflecting WGS performances (Supplementary Methods) were calculated for 52 patients and compared (Wilcoxon signed rank test). The read median insert size (Supplementary Figure S3A), aligned ratio of pass filter reads (Supplementary Figure S3B), and ratio of chimeric pairs (Supplementary Figure S3C) were significantly poorer in FFPE than FF specimens (*p* = 1.798e^− 09^, 3.618e^− 09^, and 1.330e^− 08^, respectively). In addition, FFPE samples were characterized by an important increase of AT drop out, which was significantly different from FF (*p* = 1.804e^− 09^) and a CG drop out significantly lower than FF (*p* = 1.804e^− 09^) (Supplementary Figure S3D, E, more sequencing statistics in Supplementary Table S5). The mean sequencing depth was 77 × (50–100 ×) for FFPE samples and 93 × (78–122 ×) for FF samples and was variable across chromosomes (Supplementary Figure S4). FFPE samples presented a lower proportion of regions reaching the targeted depth of 70 × : 0.351 versus 0.782 for the FF samples (Figure 1a). The uniformity of coverage, crucial for optimal variant detection (Supplementary Methods), was calculated taking the standard deviation of read coverage to measure extreme high and low signals (Figure 1b). The median standard deviation was between 1.1 × and 5.8 × higher in FFPE than matching FF (*p* < 2.2e^− 16^) meaning sequencing coverage was less uniform in FFPE data.

Purity determined by visual assessment of pathologists and determined from sequencing data showed a statistically significant positive correlation (Supplementary Figure S5, Supplementary Table S3). For 24% of cases, the estimated purity was > 40% tumor content by visual assessment and was <40% by sequencing.

### Different numbers of somatic SNVs and indels detected in FF and FFPE samples and variants agreement dependent upon the variant caller

Firstly, SNV and indel data sets of all 52 patients from FFPE and FF data were compared using several variant callers (Supplementary Table S6). Globally, somatic SNVs were increased in FFPE (Supplementary Figure S6A).

**Figure 2:**
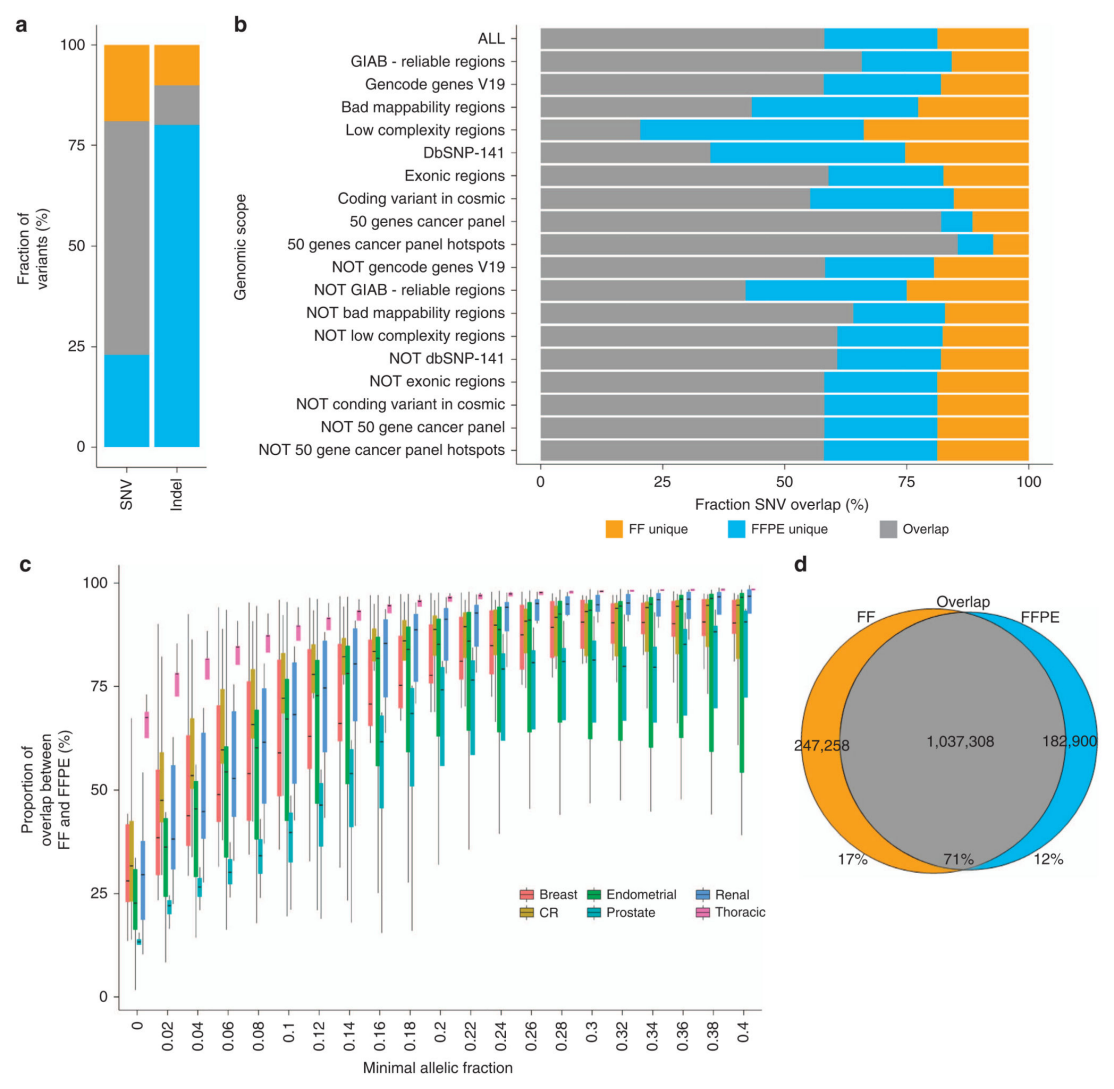
Somatic SNVs and small insertions and deletions detection and their respective overlap between FF and FFPE samples. (**a**) Agreement between FF and FFPE samples of somatic SNVs and indels represented in percent. The three sections represent the FF-unique, FFPE-unique, and FF–FFPE overlap variants. (**b**) Overlap between FF and FFPE samples of somatic SNVs in different scopes of the genome showing the fraction of variant unique in FF, unique in FFPE, and in both FF and FFPE (the different regions are detailed in Supplementary Table S8). (**c**) Evolution of the proportion of SNVs in common in FF and FFPE samples when variants with low allelic fractions are filtered from the data set for each tissue type: renal (*N* = 14), CR (*N* = 12), prostate (*N* = 4), breast (*N* = 10), endometrial (*N* = 7), and thoracic (*N* = 5). (**d**) Agreement in SNVs between FF and FFPE samples. The following variants were considered for this plot: variants in reliable regions (Genome in a Bottle regions) where the depth needed to be at least 70 × at the position of the variant for both FF and FFPE samples and the allelic fraction needed to be at least 0.067 in one of the two samples (see Supplementary Figures S12 and S19 for details). CR, colorectal; FF, fresh-frozen sample; FFPE, formalin-fixed, paraffin-embedded sample; GIAB, Genome in a Bottle; SNV, single-nucleotide variant.

FF and FFPE data sets were compared to identify variants detected either only in FF or only in FFPE sample data (“FF unique” and “FFPE unique”) and variants detected in both samples (“FF–FFPE overlap”) (Supplementary Material). A similar number of SNVs was discovered in the FF–FFPE overlap for Mutect (2.08 million) (Figure 2a) and Strelka (1.95 million), but different in proportions of agreement (58 vs. 19%) (Supplementary Figure S6B). The high number of FFPE-unique SNVs detected by Strelka considerably reduced the positive predictive value (0.21) while presenting the highest sensitivity at 0.77 (Supplementary Table S7).

Variants detected in different regions of the genome were also studied (Supplementary Table S8). A high FF–FFPE agreement was detected in reliable regions (Genome in a Bottle^31^) whereas the agreement was lower in regions of low complexity (Figure 2b).

Regarding indels, Shimmer detected more in FF samples (Supplementary Figure S6C), whereas Strelka detected more indels in FFPE (Figure 2a) leading to different FF–FFPE overlap and conflicting sensitivity and positive predictive value patterns (Supplementary Figure S6D,Supplementary Table S7). However, the number of high-risk variants (indels, start and stop codon losses and gains) was not increased in FFPE samples compared to FF (Supplementary Figure S7).

With respect to tissue types, thoracic presented more SNVs than other tissues (Supplementary Figure S8) and the highest proportion of agreement between FF and FFPE with all variant callers (Supplementary Figure S9A,B).

Finally, the proportion of C > T base substitutions (known to be enriched in FFPE specimen sequencing data that underwent deamination) was higher in the data set produced by Strelka and only to a lesser degree in data sets from other tools (Supplementary Figure S10A–D). This increase in C > T mutations observed in the Strelka data set was correlated with an increase of intergenic variants (Supplementary Figure S11).

### Tumor heterogeneity and sampling heterogeneity explained differences between FF and FFPE samples

Fifty-two percent of variants were identified in the FF–FFPE overlap, 21% were in the FF-unique data set, and 27% were in the FFPE-unique data set (Supplementary Figure S12A). Somatic SNVs unique in FF or FFPE samples were investigated. The FFPE-unique data set presented a median proportion of C > T substitutions of 24.2% (15.8–42.3), which was lower than in the overlap data set (36% (17–53.9)), confirming FFPE-unique variants were not deamination artifacts (Supplementary Figure S13A). The mutational spectrum analysis showed a higher C > A/G > T substitution rate in FFPE-unique mutations (Supplementary Figure S13B). The allelic fractions (AFs) of FF-unique and FFPE-unique variants highlighted the presence of low-level variants (Supplementary Figure S12B). This data clearly suggested that intratumor heterogeneity (variants with low allelic fractions that are truly different between FF and FFPE tissue specimens) or sampling heterogeneity (rare variants present in the tissue specimens but missed by sequencing because the individual molecules with that variant were not sampled) were important explanations for the differences observed in SNV calling.

To observe the effect of tumor and sampling heterogeneities, we filtered out SNVs detected at low AFs using several thresholds (Figure 2c). The SNVs overlap between FF and FFPE increased dramatically when mutations with low AFs were excluded. The amplitude of this effect varied in different tissue types. Thoracic sample data achieved a median SNV FF–FFPE overlap of 75% when filtering out variants with AFs lower than 0.02, making this tissue type the best-performing one (Supplementary Table S9). Other tissue types reached a median FF–FFPE overlap of 75% only if variants with AFs lower than 0.12 or 0.22 were removed, depending on tissues.

SNVs detected in different tissue types were explored further (Supplementary Figure S14A–C, Supplementary Figure S15A,B, Supplementary Figure S16). The SNV overlap between FF and FFPE samples was found to correlate with several metrics (Supplementary Figure S17A–F), but particularly with the distribution of allelic fractions of somatic SNVs: the higher the median allelic fractions, the higher the SNV overlap between FF and FFPE samples (Supplementary Figure S17G, Supplementary Figure S18). In our cohort, most thoracic samples presented a high distribution of allelic fractions and therefore a high SNV overlap between FF and FFPE samples (4 of 5), although this was not confined to this tissue type (3 other cases involved) (Supplementary Figure S17G) and did not correlate with assessed tumor purity.

Finally, to limit the effect of tumor heterogeneity and sampling heterogeneity, variants underpowered in matching FF or FFPE samples were filtered from the data set using a binomial distribution model (Supplementary Figure S19): the depth set was > 70 × at the position of the variant for both FF and FFPE samples and the allelic fractions > 0.067 in one of the two samples. With this approach, the SNV agreement between FF and FFPE samples increased from 52 to 63% when screening the whole genome (Supplementary Figure S12C) and 71% when selecting variants in reliable regions (Genome in a Bottle regions, representing 69% of the genome) (Figure 2d).

### Somatic mutations in cancer driver genes accurately detected from FFPE data sets

When considering all SNVs and indels, 90% of cancer-associated genes from the COSMIC cancer gene census^28^ were found more frequently mutated in the FFPE data set than FF data set (Supplementary Figure S20A). However, when considering the most clinically relevant types of mutation defined as functional mutations (exonic: missense, stop, frameshift, and in-frame indel mutations and splicing variants) > 99% of genes were not more mutated in FFPE than FF data sets (Supplementary Figure S20B).

In addition, 73 variants identified in 207 targets in 46 clinically relevant genes were validated in FF and FFPE using the Ion AmpliSeq Cancer Hotspot Panel v2 (Thermo Fisher Scientific), an alternative method to the Illumina technology (Supplementary Table S10). When using the panel as gold standard, the sensitivities of WGS using FF and FFPE were found to be 0.86 and 0.82, respectively, and the positive predictive values were both 1. Of the 10 variants missed in FF and 11 in FFPE by WGS, 8 had an AF <0.1 in the panel data (Supplementary Table S11). After inspection of WGS data in the Integrative Genomics Viewer,^32^ a deletion in *VHL* was confirmed in FF and FFPE data as well as one *TP53* deletion in FFPE data.

### Copy-number intensity signal was noisy in the FFPE data set

**Figure 3:**
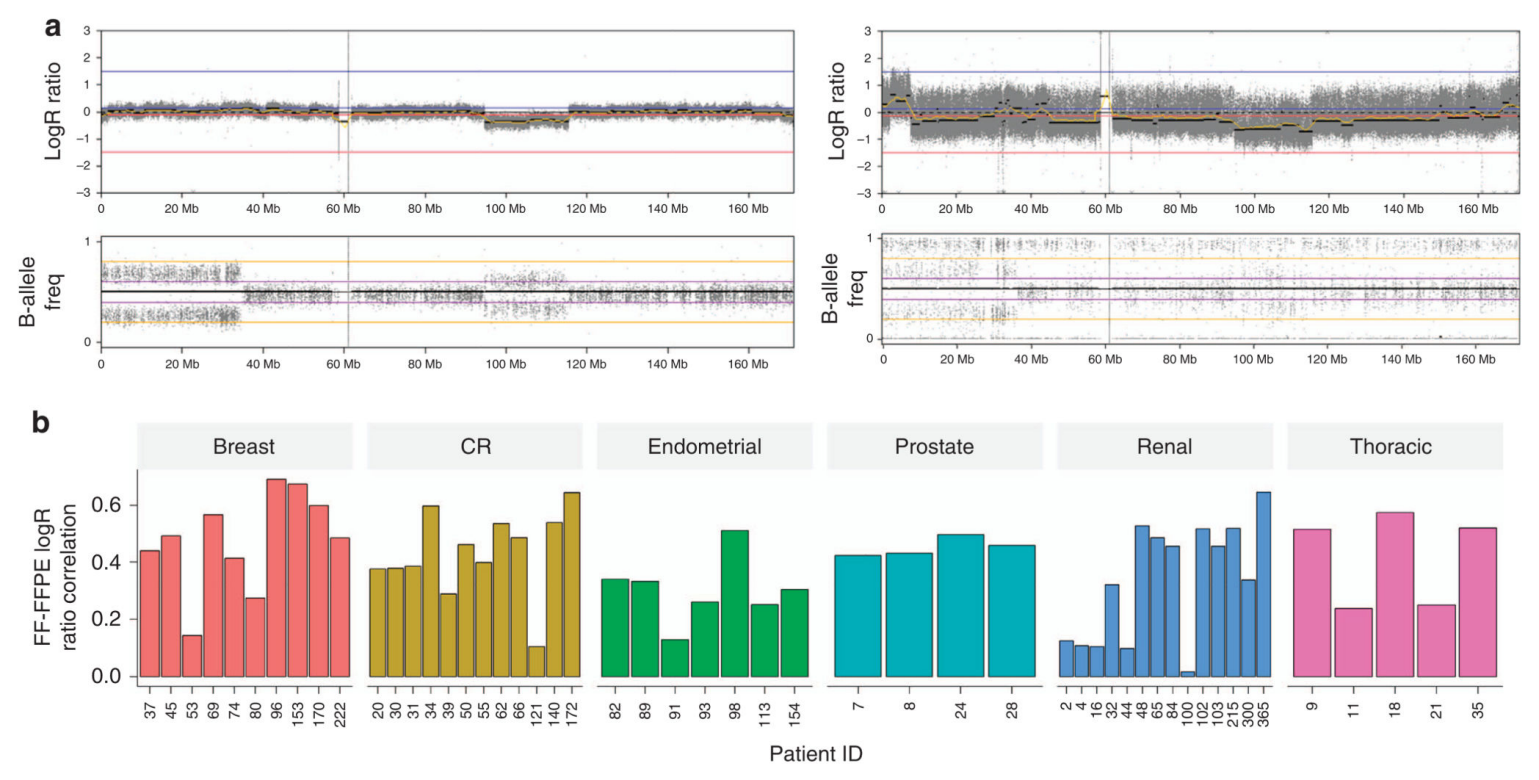
Somatic CNA detection. (**a**) Detection illustrated by a representative example of the chromosome view in Nexus Copy Number showing CNAs across chromosome 6 for case 039 in the FF sample and FFPE sample. Log_2_ ratio (LogR) shows increase in intensity for genome amplifications and decrease in intensity for deletions, and the corresponding B-allele frequency plots show variation of the median signal for loss of heterozygosity. For each of the two samples, the top panel represents the Log_2_ ratio and the bottom panel shows the B-allele frequency. (**b**) Distribution of Spearman correlation coefficient of FF Log_2_R and FFPE Log_2_R showing the agreement between FF and FFPE. A high correlation coefficient represents a high agreement between FF and FFPE samples to differentiate change in copy-number intensity signal. B-allele freq, B-allele frequency; CNA, copy-number alteration; CR, colorectal; FF, fresh-frozen sample; FFPE: formalin-fixed, paraffin-embedded sample; Log_2_R, Log_2_ ratio.

Copy-number alterations (CNAs) were also evaluated. Log_2_ ratio (Log_2_R), showing increase intensity for genome amplifications and decrease intensity for deletions, and the corresponding B-allele frequency plots, showing variation of the median signal for loss of heterozygosity, for tumor-normal pairs were compared for FF (Figure 3a) and matching FFPE samples (Figure 3b) (FF-GL independently to FFPE-GL). This example illustrated that the data points are highly noisy in FFPE samples, characterized by a larger spread of the Log_2_R values. The signal seemed to follow a wavy pattern. The median Spearman correlation coefficient of Log_2_R values was 0.435, and the highest correlation was achieved by case 96 (*r* = 0.69) (Supplementary Figure S21A–F, Figure 3c). The variant caller could not detect copy-number losses and gains reliably in FFPE samples because of the highly disrupted signal, and visual inspection was not possible due to the waviness of the Log_2_ ratio.

### Optimization of FFPE DNA extraction improved alignment metrics and detection of SNVs, indels, and CNAs

To improve coverage uniformity and therefore CNA detection we improved DNA integrity of FFPE samples. From the FFPE sample preparation and DNA extraction, 33 preanalytical variables were reviewed and we focused on the DNA extraction process. We hypothesized that modifying the de-crosslinking step would yield less fragmented nucleic acids enriched in double-stranded DNA molecules. Five illustrative cases from the 52 trios analyzed previously were selected (Supplementary Table S1). New FFPE cores were collected depending availability to test two commercially available DNA extraction kits, four de-crosslinking temperatures, two incubation times, and two salt concentrations (Supplementary Table S2).

Comparisons of sequencing alignment metrics were made to estimate the potential improvement in FFPE samples. Overall they were equivalent or improved in the experimental FFPE data sets compared with the initial FFPE data sets. In particular, AT dropout (Supplementary Figure S22A), global sequencing coverage, and coverage uniformity were improved in all experimental FFPE compared with initial FFPE data sets (Supplementary Figure S23A,B). Regarding SNVs and indels, the overlap between FF and experimental FFPE data sets was higher than in FF and initial FFPE data sets (Supplementary Figure S22B), except one condition for patient 004.

**Figure 4:**
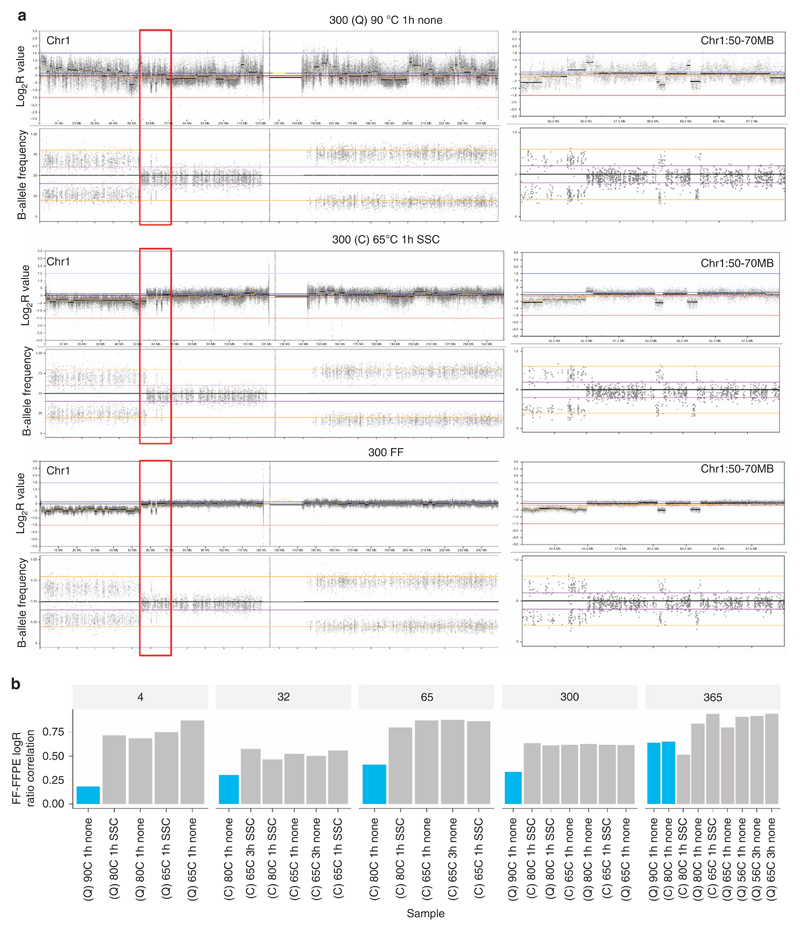
CNA detection improvement using different DNA extraction conditions from the same FFPE sample. (**a**) CNA detection improvement visualization for case 300. The left panels show the Log_2_R values and B-allele frequencies plotted for chromosome 1; the right panels show a zoomed-in view of the region in the red box (chr1:50000000–70000000) presenting two small deletions on chromosome 1. (**b**) Distribution of Spearman correlation coefficient of FF Log_2_R and FFPE Log_2_R. The experimental conditions are described in the following order: DNA extraction kit/reverse crosslinks temperature/reverse crosslinks incubation time/addition of buffer. Blue bars denote FFPE samples prepared according to the manufacturer’s instructions, gray bars represent FFPE samples prepared by optimizing the reverse crosslinking step. 300 (QC) 90 °C 1 h none is the FFPE sample from case 300 extracted with Qiagen kit (de-crosslinking conditions 90 °C for 1 h without any additional buffer); 300 (C) 65 °C 1 h SSC is the FFPE sample from case 300 extracted with Covaris kit (de-crosslinking conditions 65 °C for 1 h with additional SSC buffer); and 300 FF is the FF sample from case 300. C, Covaris kit; CNA, copy-number alteration; FF, fresh-frozen sample; FFPE, formalin-fixed, paraffin-embedded sample; Q, Qiagen kit; SSC, saline sodium citrate.

CNA detection improvement was assessed by visualization (Figure 4a) and calculation of correlation coefficients of Log_2_R between FF and experimental FFPE samples (Figure 4b, Supplementary Figure S24). Correlation coefficients were greatly improved for all experimental FFPE samples and were above 0.85 for three of five patients (patients 004, 065, and 365). These results confirmed that WGS data from poor-quality FFPE samples can be improved to enable more accurate detection of small and large mutations.

### Clinical report from optimized FFPE samples comparable to that from FF samples

Clinical whole-genome reports, including somatic SNVs, indels, and CNAs, were generated for the five patients using the optimized experimental FFPE data. These were compared with an independent analysis of the FF data (Supplementary Table S12). Forty alterations were classified as tier 1 and clinically actionable (Materials and Methods, Supplementary Methods), 64 as tier 2, and 36 as tier 3 across the five patients (Table 1). Of these alterations, 98% tier 1, 86% tier 2, and 78% tier 3 were reported in both FF and FFPE. Twelve variants were detected only in the FFPE data; all were SNVs and indels and 7 of 12 had an AF lower than 0.13. Six variants, of which three were CNAs, were detected only in FF tissue data. The remaining 51 CNAs were detected from both FFPE and FF, confirming the improved ability to detect CNAs from optimized FFPE samples.

## Discussion

We have carried out the largest study to date generating and comparing clinical WGS of cancer specimens obtained from FFPE material and matching FF samples, to answer the crucial question of whether WGS can be reliably introduced in the clinic.

Of the 184 cases recruited, a full trio (GL, FF, and FFPE) was obtained for 52, which clearly demonstrated the challenges of collecting FF tissues prospectively in routine clinical diagnostics and the DNA QC failure rates for nonoptimized FFPE tissues. Although FF samples clearly remain the optimal source of tumor DNA for WGS, limited availability is a barrier to the widespread application of WGS in clinical practice. The success rate of library preparation from FFPE DNA was much higher than previously reported^15^ (80 vs. 29.5%), due to fast processing from surgery to DNA extraction (6 months or less), and adapting the amount of input DNA based on DNA QC data (ΔCq assay, see the Supplementary Material).

Sample purity assessment by computational method generally calculated lower tumor purity than pathologist visual assessment of the specimen. Therefore initial assessment of purity of the tissue and rigorous purity inclusion criteria are crucial to ensure a sufficient detection power of subclonal variants.

Coverage depth was nonuniform alignment metrics were suboptimal for FFPE data, as described in multiple studies.^15^,^19^,^22^,^33^ However, our results were influenced by lower stringency of fragment size selection and the polymerase chain reaction step included in the FFPE library preparation method (see Materials and Methods) to improve library yield.

Unlike most previous studies focusing on GL variants,^14^,^16^,^17^,^19^–^22^,^33^–^36^ we were able to compare the detection of somatic variants between FF and FFPE data. Our results demonstrated a high discrepancy between these data sets dependent on the analytical tool set employed, validating previous observations.^36^,^37^ Consistent with previous reports, this was due to the presence of low-AF variants^17^ caused by intratumor heterogeneity and sampling heterogeneity leading to wrongly calling false-negative and false-positive variants in FFPE data (even though efforts were made to minimize this effect by collecting FF and FFPE samples from adjacent region of tissue blocks). At best, after power calculation and filtering, the somatic SNVs agreement reached 71% with 12% of variants detected only in FFPE and 17% of variants missed in FFPE. Although this result would be insufficient in a clinical setting most of these variants are found in noncoding regions, which are not clinically relevant to date. WGS coverage is limited due to cost and this technique cannot be as sensitive as targeted approaches to detect low subclonal variants (AF <0.1). Indeed, in our tumor data sets, nine clinically relevant variants with AF <0.1 called by the AmpliSeq cancer panel were not detected in either FF or FFPE tissue samples. In future years, the reduction in costs will allow for higher depth and a further increase in WGS sensitivity.

Our study also highlighted poor concordance between FF and FFPE somatic variants in regions of sequence complexity and regions with reduced read mappability. In Genome in a Bottle regions (representing 69% of the genome) higher concordance was observed demonstrating that WGS data generated from FFPE-extracted DNA presents advantages compared with whole-exome sequencing, restricted to coding regions.

Somatic CNA detection was investigated. A limitation of our approach was to combine coverage data from tumor with normal coverage data derived from blood DNA samples. It resulted in a fluctuation of Log_2_R values. To increase CNA detection accuracy in FFPE samples, we optimized the de-crosslinking step during DNA extraction. This improved coverage uniformity, particularly in AT-rich genomic regions, and resulted in calling independently 51 of 54 CNVs that had been identified in corresponding FF samples. However, visual inspection and manual curation was necessary, which is feasible in diagnostics where sequencing data is analyzed for a single patient at the time but can be challenging for cohort analyses. New statistical and bioinformatics tools that take specific features of FFPE-derived cancer sequencing data into account (such as shorter fragment lengths with nonuniform coverage) and that call CNVs and other types of structural variants with greater confidence are therefore required.

Overall our clinical reporting from FFPE was successful with 98% of clinically actionable variants (tier 1) identified.

All samples underwent “real-life” routine processing for diagnostics to preserve tumor architecture. However, there is currently no standardization of routine diagnostic FFPE processing and the preparation varies considerably depending on tissue types and sizes and according to different laboratories. These variations in the preparation lead to different DNA alterations likely to require different types of optimization, making the widespread application of FFPE-derived DNA for WGS an even greater challenge.

In conclusion, this pilot study for the 100,000 Genomes Project represents the first prospective WGS study of cancer patients comprehensively comparing results from FF and paired FFPE specimens collected in a routine clinical environment. Despite the significant shortcomings of FFPE-derived WGS data, we demonstrate that optimization of the DNA extraction process combined with careful bioinformatics analysis and visual data inspection allows confident SNV/indel and CNV calling of clinically relevant variants for diagnostic purposes. Our results support the use of optimized FFPE cancer samples as an alternative source of DNA for WGS cancer diagnostics if FF specimens are not available.

## Supplementary Material

Supplementary material is linked to the online version of the paper at http://www.nature.com/gim

1

2

3

## Figures and Tables

**Table 1:** SNV, indel, and CNA overlap of clinical report between FF and FFPE

Sample	Tier	Total	Unique FF	Unique FFPE	Overlap	% Overlap
4	1	6 (2/4)	1 (0/1)	0 (0/0)	5 (2/3)	83 (100/75)
4	2	8 (4/4)	1 (1/0)	0 (0/0)	7 (3/4)	88 (75/100)
4	3	2 (2/0)	0 (0/0)	1 (1/0)	1 (1/0)	50 (50/NA)
32	1	4 (2/2)	0 (0/0)	0 (0/0)	4 (2/2)	100 (100/100)
32	2	17 (17/0)	1 (1/0)	3 (3/0)	13 (13/0)	76 (76/NA)
32	3	7 (5/2)	1 (0/1)	3 (3/0)	3 (2/1)	43 (40/50)
65	1	11 (1/10)	0 (0/0)	0 (0/0)	11 (1/10)	100 (100/100)
65	2	12 (10/2)	0 (0/0)	1 (1/0)	11 (9/2)	92 (90/100)
65	3	11 (7/4)	1 (0/1)	1 (1/0)	9 (6/3)	82 (86/75)
300	1	8 (2/6)	0 (0/0)	0 (0/0)	8 (2/6)	100 (100/100)
300	2	16 (14/2)	0 (0/0)	2 (2/0)	14 (12/2)	88 (86/100)
300	3	6 (6/0)	0 (0/0)	1 (1/0)	5 (5/0)	83 (83/NA)
365	1	11 (2/9)	0 (0/0)	0 (0/0)	11 (2/9)	100 (100/100)
365	2	11 (8/3)	1 (1/0)	0 (0/0)	10 (7/3)	91 (88/100)
365	3	10 (4/6)	0 (0/0)	0 (0/0)	10 (4/6)	100 (100/100)
Total	1	40 (9/31)	1 (0/1)	0 (0/0)	39 (9/30)	98 (100/97)
Total	2	64 (53/11)	3 (3/0)	6 (6/0)	55 (44/11)	86 (83/100)
Total	3	36 (24/12)	2 (0/2)	6 (6/0)	28 (18/10)	78 (75/83)

CNA, copy-number alteration; FF, fresh frozen; FFPE, formalin-fixed, paraffin-embedded; NA, not applicable (no variant detected in this category); SNV, single-nucleotide variants. Total (somatic SNV+indel/somatic CNA).
