# Supplementary material for: Clinical whole-genome sequencing from routine formalin-fixed, paraffin-embedded specimens: pilot study for the 100,000 Genomes Project
Source: Genet Med. Author manuscript; Available in PMC 2019 May 16. (PMC6520241; doi:10.1038/gim.2017.241)
Supplement: 1 [file EMS82826-supplement-1.docx]

**Supplementary Materials and Methods for:**

**Clinical whole genome sequencing from routine Formalin-fixed paraffin embedded (FFPE) specimens: pilot study for the 100,000 Genomes Project**

Pauline Robbe *et al.*

## **Sample Collection and Processing**

The tissue samples were prepared as both fresh-frozen and FFPE samples as per the usual protocol in National Health Service diagnostic laboratories depending on the tissue type. For FF specimen preparation at least one punch of 5 mm was taken and was flash frozen in liquid nitrogen. The frozen punches were weighed and embedded in OCT (Embedding medium for optimal cutting temperature). H&E sections were taken and assessed by a pathologist for percentage of viable tumour cells (out of total number of cells present) and percentage necrosis (table S3). Samples with >40% viable invasive tumour cells were suitable for the study. The remainder of the specimen was prepared as FFPE. Briefly, all samples underwent fixation in 10% formalin buffered either with sodium chloride or phosphate for routine sectioning: this was usually carried out the following day, but the optimal duration of fixation depends on specimen type and/or time of sample receipt. In most cases, the FFPE block was taken in the area surrounding the FF punch (figure S25). Pathologists assessed the FFPE slides corresponding to the block and chose a high cellularity area which is well preserved and avoided areas of necrosis if possible. All FFPE samples were stored at room temperature.

## **Nucleic Acids extraction and Quality Control**

For peripheral blood, genomic DNA samples were extracted with QIAamp DNA Mini Kit (Qiagen, Hilden, Germany) as per the manufacturer’s instructions. For FF tissues, genomic DNA was extracted from approximately 30mg using the All Prep DNA/RNA Mini Kit (Qiagen, Hilden, Germany) following the manufacturer’s protocol. For FFPE tissues, two 1 mm diameter core punches were collected from the paraffin block and DNA was extracted by the truXTRAC FFPE DNA Kit (Covaris, Woburn, MA) according to the manufacturer’s instructions for extraction using a M220 ultrasonicator (Covaris), performing an overnight incubation with proteinase K at 56°C.

Quantity and quality of the 52 matching FF and FFPE DNAs were compared using paired Wilcoxon signed rank tests and Bonferroni corrected p-values. The median time between specimen collection and DNA extraction of 66.1 days for FF samples and 56.2 days for FFPE samples was not significantly different allowing comparison of other QC metrics (p=0.526, paired Wilcoxon signed rank tests and Bonferroni corrected p-value accounting for the multiple metrics considered) (table S4). Nucleic acids were quantified with Nanodrop (Thermo Fisher Scientific, MA, USA) for quantification of all nucleic acids and Qubit dsDNA Broad Range Assay kit (Thermo Fisher Scientific, MA, USA) for quantification of double stranded nucleic acids. DNA degradation and purity were assessed by 3% agarose electrophoresis and A260/A280 Nanodrop ratio. DNA quality from FFPE samples was also assessed to determine the amount of amplifiable DNA by SYBR-green based quantitative real-time PCR assay FFPE QC PCR (Illumina, San Diego, USA) according to the manufacturer’s instructions. For each FFPE sample, a ΔCq value was calculated by subtracting its threshold cycle (Ct) value from the Ct value of a high-quality reference FF DNA sample normalised and evaluated in parallel (table S13). To pass QC, the minimum yield required was 1 µg for all FF and GL samples and 400 ng for FFPE samples with a ΔCq value above 1. Although seven out of 52 FFPE samples had a ΔCq greater than the threshold of 2.5, which is the cut-off defined by the manufacturer’s instructions libraries were prepared for all of samples.

**Nucleic acids extraction optimisation**

For the QIAamp DNA FFPE Tissue kit (Qiagen) we used deparaffinisation solution for the paraffin removal step and incubated overnight with proteinase K. TruXTRAC FFPE DNA (Covaris) extraction was performed as previously described.

## **FFPE Library Preparation**

FFPE DNA derived libraries were prepared using 250 ng DNA for samples with a ΔCq ≤ 1 and 400ng for samples with a ΔCq >1. To address known FFPE features in particular deamination, two DNA repair steps, Rep1 and Rep2, were included after DNA fragmentation, followed by end repair, size selection, adapter ligation and eight PCR cycles were performed on library samples (95°C for 3 minutes; 8 cycles of: 98°C for 20 seconds, 60°C for 15 seconds, 72°C for 30 seconds; 72°C for 5 minutes).

## **Alignment and Alignment Metrics**

Read alignment to human reference GRCh37.1 was achieved using Isaac aligner version 01.14.02.06 and version SAAC00776.15.01.27^1^. Quality control was done with Isaac Variant Caller^2^. Alignment metrics were calculated using Picard tools. Sequencing coverage metrics were calculated using an in-house coverage analysis tool and CODOC^3^. Alignment metrics such as median insert size, read PF aligned ratio, chimeric pairs ratio, AT drop-out and GC drop-out were calculated using Picard tools.

- Median insert size: median length between extremities of paired reads. The higher the better, there is a size selection during the library preparation for 350bp fragments.
- Read PF aligned ratio: fractions of sequencing reads that have a position on the reference genome. The higher the better, below 80% is indicative of poor quality sample.
- Chimeric pairs ratio: Paired reads which align to different regions or chromosomes.
- AT drop-out: a measure of how less covered regions with GC content < 50% are compared to the mean.
- GC drop-out: a measure of how less covered regions with GC content > 50% are compared to the mean.

Percent aligned to the human genome reference and High AT/GC normalised coverage indicate coverage uniformity. Insert sizes and chimeric reads values correlate with the number of potential somatic false positive variants.

It is important to highlight that these measurement are slightly dependent on the library preparation method. In order to increase library quantity, smaller fragments are included in the FFPE preparation. In addition, the PCR step specific to the FFPE library preparation can lead to some biases.

We also measured the number of bases covered at different thresholds (from less than 3 x to more than 100 x) and the uniformity of sequencing coverage by calculating the standard deviation of the sequencing depth of 100 kb genomic windows. A high value indicated a highly variable sequencing coverage directly impacting on CNA detection. Poor sequencing coverage uniformity is an issue as it leads to waste of sequencing data by over sequencing some regions of the genome and impacts the quality of variant detection with lower coverage in other part of the genome.

## **Purity assessment from WGS data**

Purity estimation was computed from Canvas^4^. The comparison of purity assessment from WGS data and visual assessment by pathologist is challenging because of the discrepancy in defining tumour purity by these two methods. The computational assessments of tumour purity using sequencing data will identify the proportion of reads with somatic variants (SNVs, CNAs, or both, depending on the algorithms) and infer the proportion of cells in the sample carrying these somatic mutations (i.e. tumour cells). The computational purity assessment is therefore dependent on sampling and the alignment performances. On the other hand, pathologists visually quantify the proportion of cells and depending on the tissue type might consider some stromal cell infiltration as part of the malignant process. Another explanation for estimation variability for this method could be that the tumour cells are bigger than stromal cells and inflammatory cells and therefore are more visible. This “overestimation” has been previously reported in another study by Viray et al^5^.

## **Motivation for SNV Variant Calling**

Variant calling was performed in an unbiased way (i.e. in FF and FFPE, independently) to efficiently assess the potential of variant calling in FFPE specimens. As most bioinformatics algorithms are developed for sequencing data originating from fresh tissue DNA, we expected diverse algorithms would give different results. Therefore, to ensure that the differences originated from the samples and not from poor performance of the algorithms on FFPE data somatic SNV detection was performed with Mutect v1.1.4^6^, Shimmer v0.1.1^7^ and Strelka 2.0.14^8^. Variants were annotated with Ensembl Variant Effect Predictor (VEP) GRCh37 release 85^9^. Variant statistics were calculated with BCFtools v1.1^10^.

## **VCF Intersect**

High confidence variants were called by intersecting datasets from Mutect, Strelka and Shimmer for SNVs, and Strelka and Shimmer for indels. They cancel the potential specific bias of each algorithm as they are detected by all three variant callers. VCF Intersect compares the output (VCF files) of variant calling pipelines by performing several steps: data cleaning (splitting complex variants, normalising chromosome names and order), including/excluding genomic regions, performing scoped comparison and generating quality metrics (summary statistics, Venn diagrams and VCF files for manual inspection or follow up computations).

## **SNV and Indel Dataset Comparisons and Investigation**

The unfiltered lists of somatic mutations from FF and FFPE sample datasets were compared directly using VCF Intersect to identify variants detected either only in FF or only in FFPE sample data (labelled as “FF unique” and “FFPE unique”) and variants detected in both samples (named “FF-FFPE overlap”). For this analysis, we calculated the sensitivity and PPV using FF sample data as the reference dataset. Somatic SNVs detected only in one sample of the pair were investigated further using the dataset produced with Mutect as it offered the best balance between sensitivity, PPV and number of SNVs detected in both FF and FFPE DNA samples and investigated the presence of potential deamination artefacts and the AF directly computed from the BAM alignment files using vcfBAFAnnotator. The variant detection limits for clinically relevant somatic SNVs and indels using FFPE data were tested using the Strelka variant datasets as these showed the best sensitivity in both SNV and indel detection.

## **Ampliseq Cancer Hotspot Panel and WGS Variant Validation**

Sequencing libraries were generated from 5 ng input DNA using the AmpliSeq Cancer Hotspot panel according to manufacturer’s instructions (Thermo Fisher Scientific, MA, USA) and run on an Ion PGM System (Thermo Fisher Scientific, MA, USA). The software Ion Reporter was used to report the variants and classified according to the methods described by Veenstra et al ^11^ and Khoury et al ^12^. All FF samples were processed by targeted sequencing. However, due to lack of DNA, FFPE samples were sequenced on the panel only if a variant was not detected in the WGS data of the FFPE sample and was detected in FF by both methods (targeted panel and WGS). Variants could not be verified for the FFPE samples with the targeted panel for patients 030 and 172. In these cases, possible tumour heterogeneity could explain the presence of such variants in FF and absence from the matching FFPE sample. Therefore, a total of 69 variants were evaluated in FF samples and 61 were evaluated in FFPE samples. All variants were visualised in the integrative genomics viewer (IGV)^13^. The number of reference reads and altered reads were directly computed from the variant caller. In case a variant was not found by the variant caller, such measurements were derived from IGV visualisation.

## **CNA Calling Using Nexus Discovery Edition 7.5**

Somatic CNAs were called with Nexus Discovery Edition 7.5 (BioDiscovery Inc., El Segundo, California, USA) using Log2R and B-allele frequency data extracted from the read alignments. The Log_2_R signal was extracted using the ngCGH script^14^ with a configured window size of 500 reads. B-allele frequency information was obtained from VCF files generated by Platypus 0.5.2^15^.

## **Clinical Reporting**

Alterations found in exons and UTRs of 903 genes (from COSMIC cancer gene census referencing 600 genes, and genes of the Renal Cell carcinoma and PI3K-Akt signalling KEGG pathways) were analysed further for clinical reporting. Variants were annotated using VEP GRCh37 release 85^9^ to filter out common polymorphisms from ExAC^16^ and 1000 Genomes project^17^. The following alterations types were considered: SNVs, indels, CN gains and losses and copy neutral loss of heterozygosity (cnLOH); in exonic regions (Frameshift and in-frame insertions and deletions, missense variants and stop gains and losses) in 3 and 5 prime untranslated region (UTR) and in splicing regions.

These variants were divided into tiers (also described in figure S26):

- Tier 1 included variants in actionable genes in any cancers (meaning such variants detected can influence the patient’s treatment, survival or to gain access to a clinical trial, as described in clinicaltrial.gov or mycancergenome.org). These variants have to follow the same mechanism of action (MOA) as described in the literature (gain of function or loss of function of the protein affected from cancer.sanger.ac.uk/cosmic, cbioportal.org and cancer-genetics.org/) and in the case of a cnLOH, it needs to be associated with a mutation.
- Tier 2 included cnLOH not associated with a mutation in an actionable gene known to be affected by loss of function. This tier also included other types of mutations in actionable genes affecting the same MOA as described in the literature, susceptible to alter the protein (copy number changes, stop codon gains and losses and indels) or recurrently found mutated in the cancer genome atlas (TCGA) ^18,19^. Finally, tier 2 also included non-actionable variants if the variants presented the same MOA as described in the literature. In case the MOA was not known in the literature, the variant had to be susceptible to alter the protein (copy number changes, stop codon gains and losses and indels) or recurrently found mutated in TCGA and affecting a protein domain.
- Tier 3 gathered actionable genes with variants not following the same MOA as that described in the literature. In case the MOA was unknown in the literature, variants were reported in tier 3 if they were missense variants or in UTRs, not recurrently found mutated in TCGA or not affecting a protein domain. Finally, non-actionable variants, with unknown MOA in the literature, recurrently found mutated in TCGA but not affecting a protein domain, were also classified as tier 3.

## **Statistical Analysis**

Continuous data comparisons were performed using t-tests for normally distributed variables and Wilcoxon signed rank tests for asymmetrical distributions. Multiple testing adjustments of p-values were performed using a Bonferroni correction. In our statistical approach we assumed different variances between the two groups tested. Data transformation of ratios was performed using a Blom transformation. Linear relationships were calculated with Spearman correlation coefficients r. SNV and indel detection sensitivity was calculated as follow: number of variants detected in FF and FFPE / number of all variants detected in FF. The positive predictive value (PPV) was calculated as follow: number of variants detected in FF and FFPE / number of all variants detected in FFPE.

## **References**

1. Raczy C, Petrovski R, Saunders CT, et al. Genome analysis Isaac : ultra-fast whole-genome secondary analysis on Illumina sequencing platforms. *Bioinformatics*. 2013;29(16):2041-2043. doi:10.1093/bioinformatics/btt314.

2. Illumina. Isaac Genome Alignment and Isaac Variant Caller. 2014. https://www.illumina.com/documents/products/whitepapers/whitepaper_isaac_workflow.pdf.

3. Popitsch N. CODOC: Efficient access, analysis and compression of depth of coverage signals. *Bioinformatics*. 2014;30(18):2676-2677. doi:10.1093/bioinformatics/btu362.

4. Ivakhno S, Roller E, Colombo C, Tedder P, Cox AJ. Canvas SPW: Calling De Novo Copy Number Variants In Pedigrees. *bioRxiv*. March 2017. http://biorxiv.org/content/early/2017/03/29/121939.abstract.

5. Viray Dr. H, Li K, Long TA, et al. A prospective, multi-Institutional diagnostic trial to determine pathologist accuracy in estimation of percentage of malignant cells. *Arch Pathol Lab Med*. 2013;137(11):1545-1549. doi:10.5858/arpa.2012-0561-CP.

6. Cibulskis K, Lawrence MS, Carter SL, et al. Sensitive detection of somatic point mutations in impure and heterogeneous cancer samples. *Nat Biotechnol*. 2013;31(3):213-219. doi:10.1038/nbt.2514.

7. Hansen NF, Gartner JJ, Mei L, Samuels Y, Mullikin JC. Shimmer: Detection of genetic alterations in tumors using next-generation sequence data. *Bioinformatics*. 2013;29(12):1498-1503. doi:10.1093/bioinformatics/btt183.

8. Saunders CT, Wong WSW, Swamy S, Becq J, Murray LJ, Cheetham RK. Strelka: Accurate somatic small-variant calling from sequenced tumor-normal sample pairs. *Bioinformatics*. 2012;28(14):1811-1817. doi:10.1093/bioinformatics/bts271.

9. McLaren W, Pritchard B, Rios D, Chen Y, Flicek P, Cunningham F. Deriving the consequences of genomic variants with the Ensembl API and SNP Effect Predictor. *Bioinformatics*. 2010;26(16):2069-2070. doi:10.1093/bioinformatics/btq330.

10. Li H, Handsaker B, Wysoker A, et al. The Sequence Alignment/Map format and SAMtools. *Bioinformatics*. 2009;25(16):2078-2079. doi:10.1093/bioinformatics/btp352.

11. Veenstra DL, Roth JA, Garrison LPJ, Ramsey SD, Burke W. A formal risk-benefit framework for genomic tests: facilitating the appropriate translation of genomics into clinical practice. *Genet Med Off J Am Coll Med Genet*. 2010;12(11):686-693. doi:10.1097/GIM.0b013e3181eff533.

12. Khoury MJ, Coates RJ, Evans JP. Evidence-based classification of recommendations on use of genomic tests in clinical practice: Dealing with insufficient evidence. *Genet Med*. 2010;12(11):680-683. doi:10.1097/GIM.0b013e3181f9ad55.

13. Grever MR, Lucas DM, Dewald GW, et al. Comprehensive assessment of genetic and molecular features predicting outcome in patients with chronic lymphocytic leukemia: Results from the US intergroup phase III trial E2997. *J Clin Oncol*. 2007;25(7):799-804. doi:10.1200/JCO.2006.08.3089.

14. Sean Davis. ngCGH. 2016. http://github.com/seandavi/ngCGH.

15. Rimmer A, Phan H, Mathieson I, et al. Integrating mapping-, assembly- and haplotype-based approaches for calling variants in clinical sequencing applications. *Nat Genet*. 2014;46(November 2013):1-9. doi:10.1038/ng.3036.

16. Lek M, Karczewski K, Minikel E, et al. Analysis of protein-coding genetic variation in 60,706 humans. *bioRxiv*. October 2015. http://biorxiv.org/content/early/2015/10/30/030338.abstract.

17. Project G, Project G, Asia E, Africa S, Figs S, Tables S. An integrated map of genetic variation from 1,092 human genomes. *Nature*. 2012;135(V):0-9. doi:10.1038/nature11632.

18. Cerami E, Gao J, Dogrusoz U, et al. The cBio Cancer Genomics Portal: An open platform for exploring multidimensional cancer genomics data. *Cancer Discov*. 2012;2(5):401-404. doi:10.1158/2159-8290.CD-12-0095.

19. Gao J, Aksoy B, Dogrusoz U, Dresdner G. Integrative analysis of complex cancer genomics and clinical profiles using the cBioPortal. *Sci Signal*. 2013;6(269):1-20. doi:10.1126/scisignal.2004088.
