# Supplementary material for: Clinical whole-genome sequencing from routine formalin-fixed, paraffin-embedded specimens: pilot study for the 100,000 Genomes Project
Source: Genet Med. Author manuscript; Available in PMC 2019 May 16. (PMC6520241; doi:10.1038/gim.2017.241)
Supplement: 2 [file EMS82826-supplement-2.docx]

**Supplementary Figures for:**

**Clinical whole genome sequencing from routine Formalin-fixed paraffin embedded (FFPE) specimens: pilot study for the 100,000 Genomes Project**

Pauline Robbe *et al.*


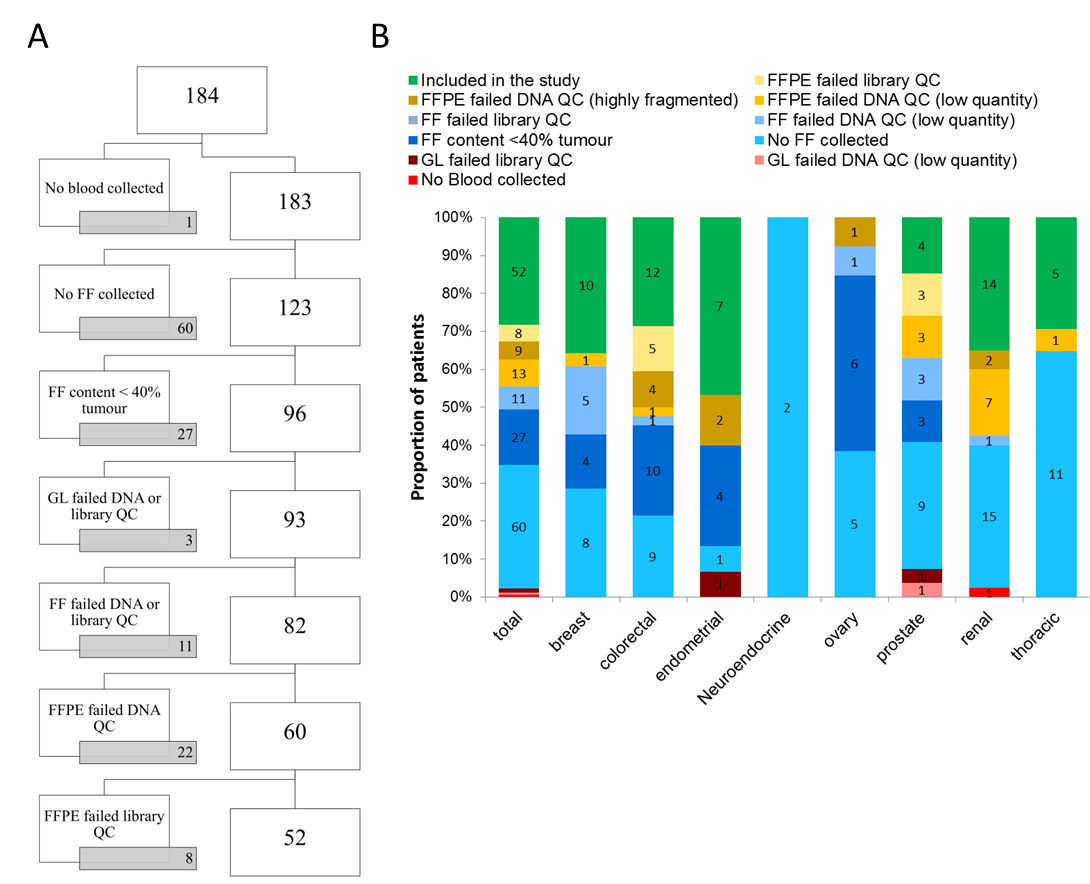


fig. S 1 Patient dropout at different quality control steps.

Summary overview of the patient dropout (A), detailed quality control dropout for each tissue type (B). Dropout was measured for sample collection, DNA quality control and library quality control. FFPE samples used were not optimised.

FF: Fresh Frozen sample, FFPE: Formalin-fixed paraffin embedded sample, QC: Quality Control, GL: germline (from Blood)


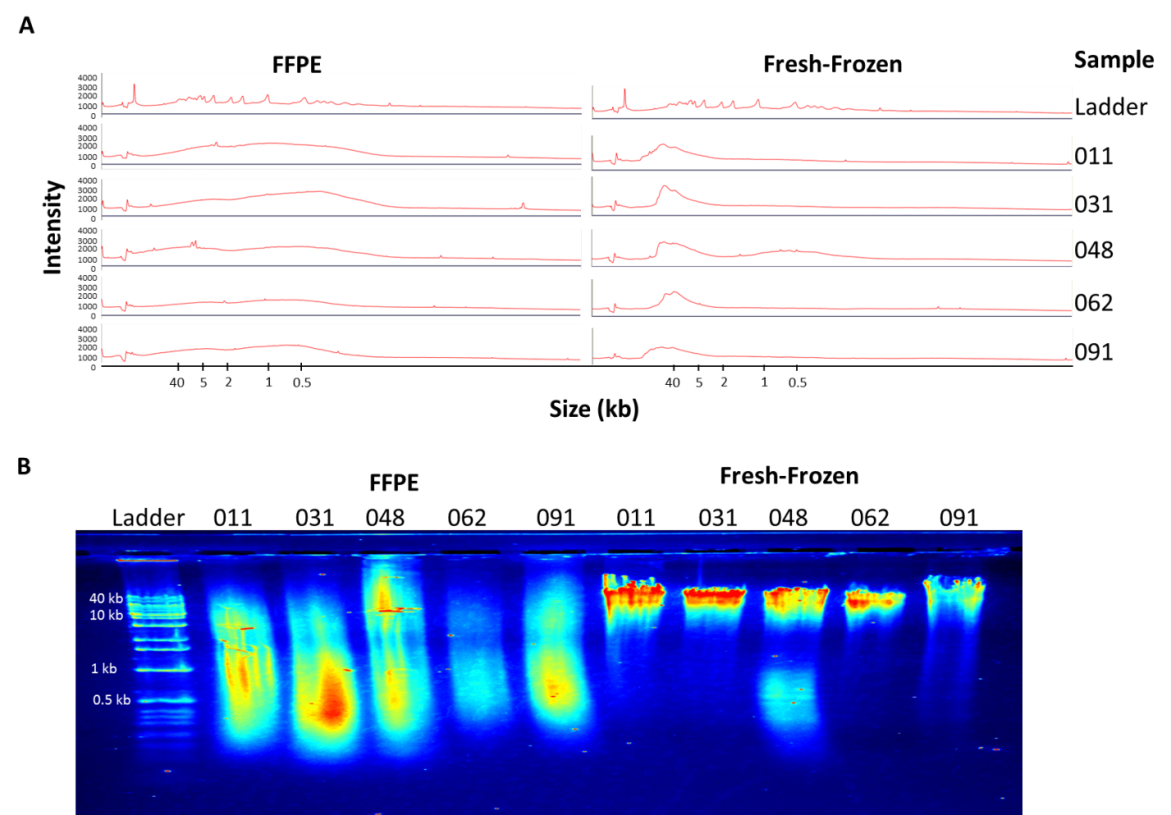


fig. S2. DNA integrity quality control.

DNA electrophoresis traces (A) and digital printout of relative intensity of DNA bands (B) of a 3% agarose gel electrophoresis result showing five representative pairs of FF and FFPE samples from the patient cohort. The ladder used was the 1 Kb DNA Extension Ladder (Thermo Fisher Scientific, MA, USA) (the FFPE sample 048 showed an ethidium bromide artefact at 10 kb).

FF: Fresh Frozen sample, FFPE: Formalin-fixed paraffin embedded sample

**fig. S3 Distribution of sequencing metrics.**

The metrics are presented as follow: median insert size in bp (A), Read PF aligned ratio (B), Chimeric pairs ratio (C), proportion of AT (D) and GC dropout (E) in percent in FF and matching FFPE samples (N=52); Wilcoxon signed rank test and Bonferroni corrected p-values. See supplementary Material and Methods for a description of the metrics

Fig. S 4 Distribution of sequencing depth per chromosome for FF and FFPE samples

Each box represents the distribution of mean coverage in 100 kb windows for each of the 52 samples. Each panel represents a chromosome (chr23=X, chr24=Y).

FF: fresh frozen, FFPE: formalin fixed paraffin embedded.


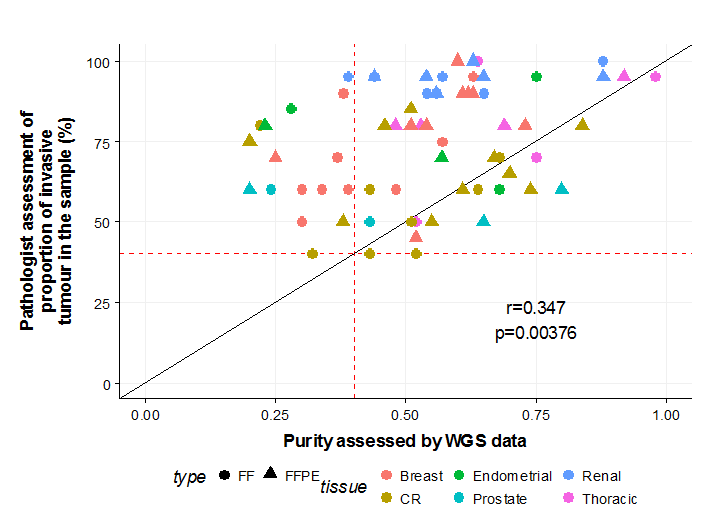


Fig. S 5 Correlation between pathologist assessment of proportion of invasive tumour in the sample and purity assessment by WGS data.

Canvas was used to compute the purity from WGS data. Correlation coefficient and p values were calculated using a Spearman's rank correlation. Pathologist visual assessment (y axis) excluded all samples with a percentage less than 40% (red dotted horizontal line). For the majority of samples the pathologist’s visual assessment gave a greater proportion of invasive tumour compared to the purity assessed by WGS data. All samples to the left of the vertical dotted line were samples with visual estimation of tumour content greater than 40% and computational assessment from WGS data less than 0.4 (32% FF samples and 15% FFPE samples).

FF: fresh frozen, FFPE: formalin fixed paraffin embedded, CR: colorectal.

**fig. S6 Somatic Single Nucleotide variants and small insertions and deletions detection and their respective overlap between FF and FFPE samples.**

Number of SNVs (A) and indels (C) reported using each variant detection method (Mutect, Shimmer, Strelka and the combination method) in FF and FFPE samples (across all samples); overlap between FF and FFPE samples of somatic SNVs (B) and indels (D) represented in percent for each variant detection method (Mutect, Shimmer, Strelka and the combination method)

FF: fresh frozen, FFPE: formalin fixed paraffin embedded, SNVs: single nucleotide variants

Fig. S 7 Distribution of the number of high-risk variants for FF and FFPE samples using different variant callers.

High-risk variants are defined by frameshift indels, inframe deletion, inframe insertions, start lost, stop gained and stop lost variants. FF and FFPE samples presented a similar number of these variants.

FF: fresh frozen; FFPE: formalin fixed paraffin embedded

Fig. S 8 Absolute count of SNVs per sample, per tissue types for FF and FFPE samples

More variants were detected in thoracic samples than in other tissues. Case 82 was excluded from this plot as it is a hypermutated endometrial case (see Table S6).

CR: colorectal; FF: fresh frozen; FFPE: formalin fixed paraffin embedded; SNVs: single nucleotide variants

**fig. S9 SNV comparison and indel comparison between matching FF and FFPE samples according to the sample tissue type.**

Measurement of overlap for somatic SNVs (A) and indels (B) represented in percent. (N=52).

CR: colorectal; Endomet.: endometrial; Prost.: prostate, FF: Fresh Frozen sample, FFPE: Formalin-fixed paraffin embedded sample, SNVs: single nucleotide variants


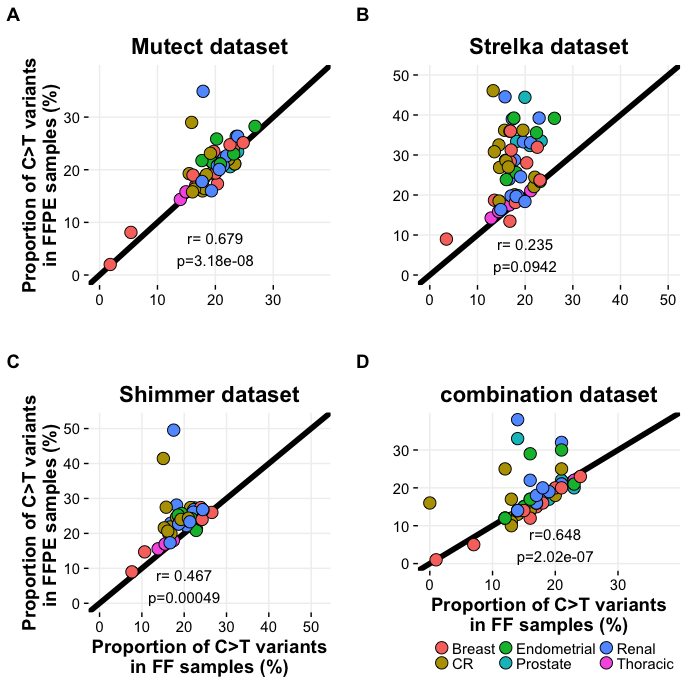


fig. S10 Correlation of proportion of C>T base substitutions between FF and FFPE samples

Correlation of proportion of C>T base substitutions (with Spearman's rank correlation) for the Mutect dataset (A), the Strelka dataset (B) the Shimmer dataset (C) and the combination dataset (high confidence variants) (D).

FF: Fresh Frozen sample, FFPE: Formalin-fixed paraffin embedded sample, CR: colorectal, Endomet.: endometrial

Fig. S 11 Distribution of number intergenic variants per sample in FF and FFPE

Intergenic variants were the only type of variants significantly increased in the FFPE Strelka dataset (paired t-test comparing Strelka and Mutect, p= 1.16e-03).

FF: Fresh Frozen sample, FFPE: Formalin-fixed paraffin embedded sample


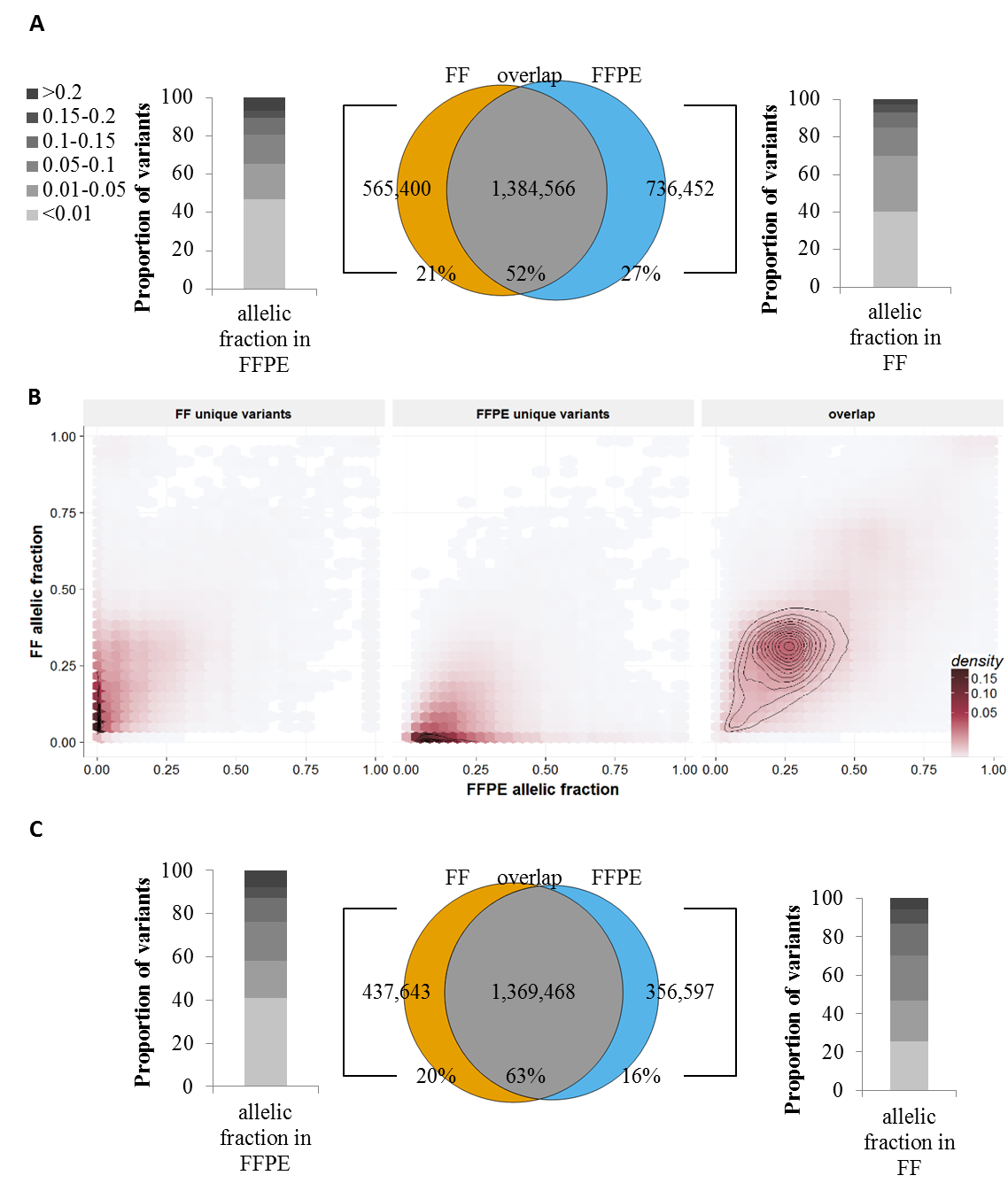


Fig. S 12 Somatic Single Nucleotide variants overlap between FF and FFPE samples

Overlap between all FF and all FFPE variants (no filters applied) (A). The allelic fractions (AFs) directly computed from the alignment data (BAM file) were examined for all genomic positions where somatic SNVs were detected in DNA from at least one of the FF or FFPE specimens of a pair. 53% of FF unique variants presented an AF in the FFPE BAM file > 0.01 (were present in the sample, but not detected by the variant caller because of lack of confidence). Also, 60% of FFPE unique presented an AF in the FF BAM file > 0.01. Therefore this analysis showed that some variants might have been misclassified in our analysis due to variant calling limitations at the sequencing depth available. Allelic fraction of FF and FFPE samples at position of somatic SNV (N=2,686,418) separated in three different groups: SNVs detected in FF only (N=565,400), SNVs detected in FFPE only (N=736,452) and SNVs detected in both FF and FFPE (N=1,384,566) (B). The shading in the left and middle panels represents variants not detected by the variant caller (VCF file), but with an allelic fraction greater than 0 in the alignment (BAM file). Overlap between FF and FFPE variants with variant not sufficiently powered were filtered out according to Fig. S13: the depth needed to be at least 70X at the position of the variant for both FF and FFPE samples and the allelic fraction needed to be at least 0.067 in one of the two samples. (C). The proportion of variant overlap was improved from 52% to 63%, by mainly removing FFPE unique variants.

FF: Fresh Frozen sample, FFPE: Formalin-fixed paraffin embedded sample

Fig. S 13 Mutation spectrum of FF unique, FFPE unique and overlap variants

Proportion of C > T or G > A substitutions per patient: variants detected are categorised into three groups: unique in FF, unique in FFPE, or found in the intersection of FF and FFPE where each data point represent the proportion of C>T/G>A mutation in a patient (N=52) (A); Comparison of the mutational spectrum between FF unique, FFPE unique and overlap variants (B).

FF: Fresh Frozen sample, FFPE: Formalin-fixed paraffin embedded sample

Fig. S 14 Distribution of variant type per tissue type in FF and FFPE samples.

A minority of variants were found in splicing regions and TF binding site (A); less than 1% of the variants were found in exonic regions and UTRs, between 1 and 3% were found in regulatory regions and non-coding variants (non-coding transcript exon variant and non-coding transcript variant) (B); the largest proportion of variants was found in intergenic and intronic regions (C).

FF: fresh frozen; FFPE: formalin fixed paraffin embedded, CR: colorectal, UTR: untranslated regions; TF: transcription factor.

Fig. S 15 Mutation spectrum for different tissue types

Comparison of the absolute number of variants and the different type of variants in different tissues and in FF and FFPE samples (A); Comparison of the mutational spectrum between different tissues and in FF and FFPE samples (B).

Endomet.: endometrial; CR: colorectal; FF: fresh frozen, FFPE: formalin fixed paraffin embedded

Fig. S 16 Distribution of allelic fractions of somatic SNVs per tissue type for FF and FFPE samples.

Endometrial and thoracic tissues presented the highest median allelic fractions.

Endomet.: endometrial; CR: colorectal; FF: fresh frozen, FFPE: formalin fixed paraffin embedded, SNVs: single nucleotide variants

Fig. S 17 Statistically significant correlation between proportion of SNV overlap between FF and FFPE samples and several metrics.

The metrics studied were: the absolute number of C>A/G>T mutations (A), the median insert size (B), the proportion of reads aligned (C), the proportion of chimeric pairs (D), the median of number of reads covered (E), the absolute number of SNVs in the FFPE sample (F) and the allelic fraction median of somatic SNVs from the FFPE sample (G). Correlation coefficient and p values were calculated using a Spearman's rank correlation.

FFPE: formalin fixed paraffin embedded, SNVs, single nucleotide variants, CR: Colorectal

Fig. S 18 Distribution of allelic fractions of somatic SNVs for FF and FFPE samples

The horizontal line is plot for y=0.237 which delineate the top 7 FFPE samples performing better regarding SNVs overlap with matching FF sample (cases 9, 11, 21, 31, 35, 82, 153).

FF: fresh frozen, FFPE: formalin fixed paraffin embedded, SNVs: single nucleotide variants

Fig. S 19 Probability of observing at least 1 read with variant using binomial distribution.

For a depth of 70X and the minimal allelic fraction detectable is 0.067 with a probability of 0.95. The vertical bar showed an allelic fraction of 0.067 and the horizontal bar showed a probability of 0.95.


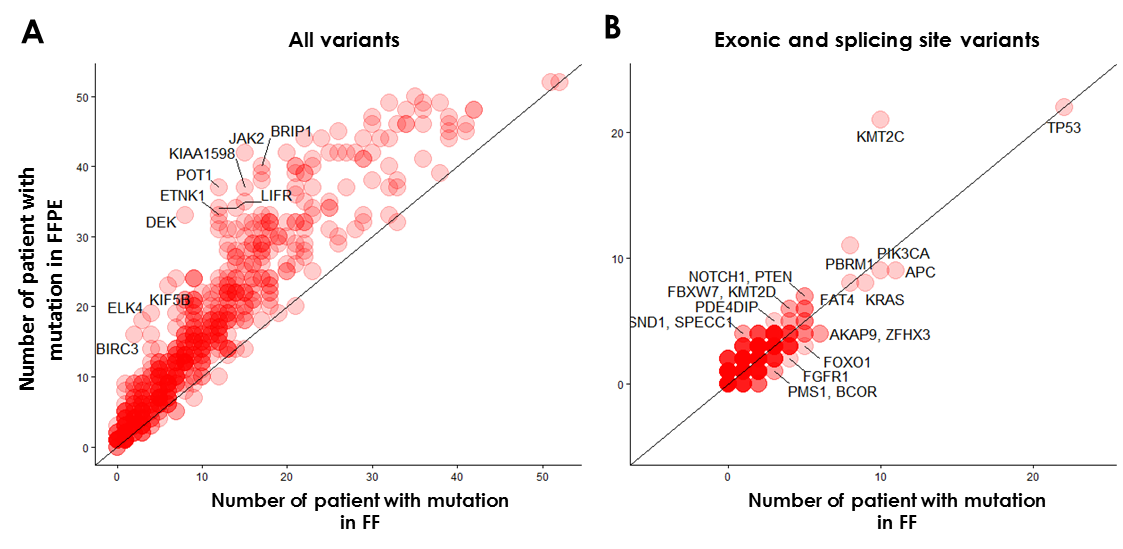


Fig. S 20 Somatic Single Nucleotide variants and small insertions and deletions comparison between FF and FFPE samples in cancer related genes.

Proportion of patients mutated in 600 genes from the COSMIC census in 52 FF samples compared with the matching 52 FFPE samples considering all SNV and indel types (A) and specifically splicing regions and exons: missense, stop, frameshift and in-frame indels (B) (N=600).

FF: fresh frozen, FFPE: formalin fixed paraffin embedded, SNVs: single nucleotide variants


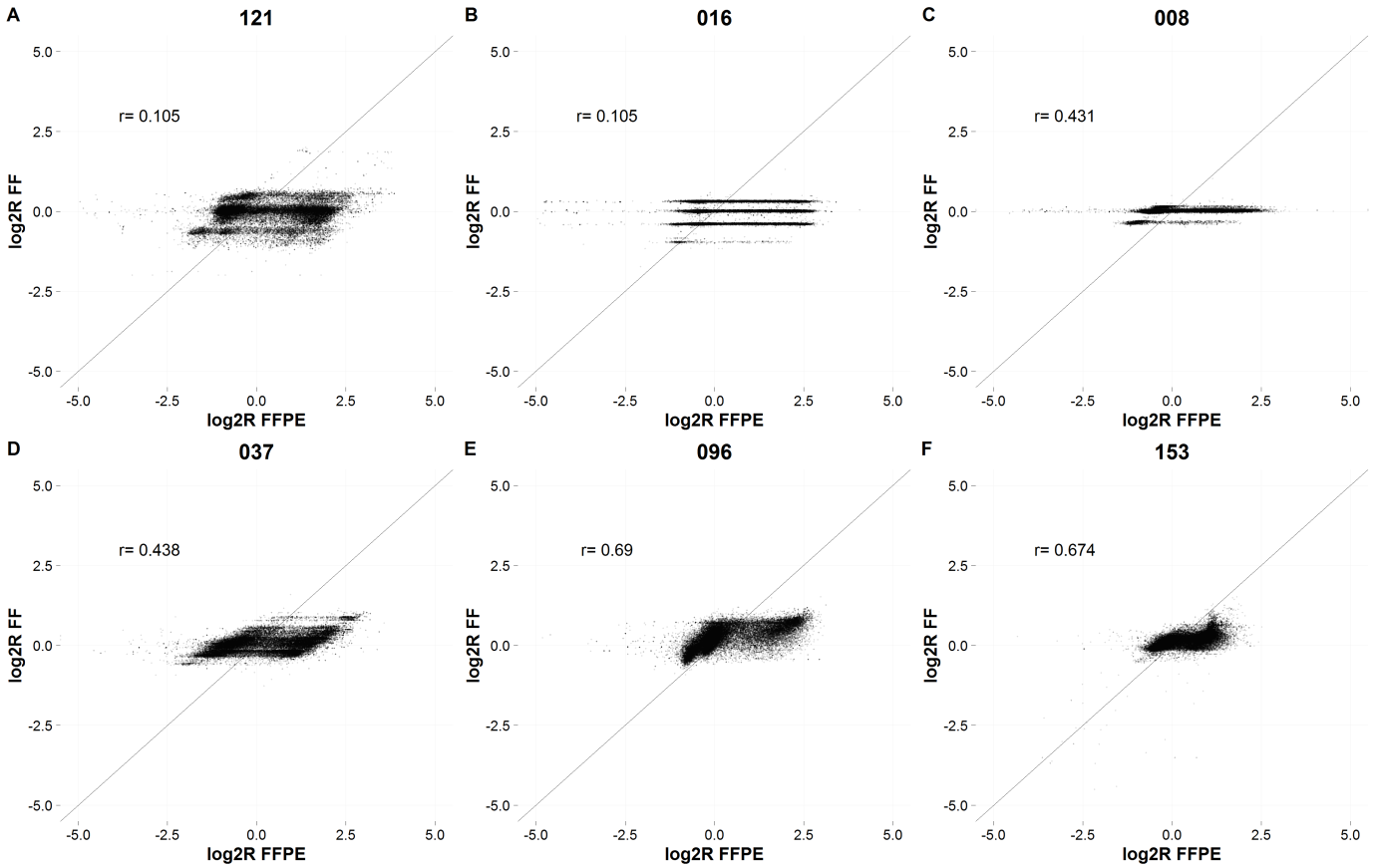


**fig. S21 Log_2_R correlation between FF and FFPE samples of six representative samples.**

Spearman correlation coefficients are indicated in each plot.

FF: fresh frozen, FFPE: formalin fixed paraffin embedded, log2R: log2 ratio


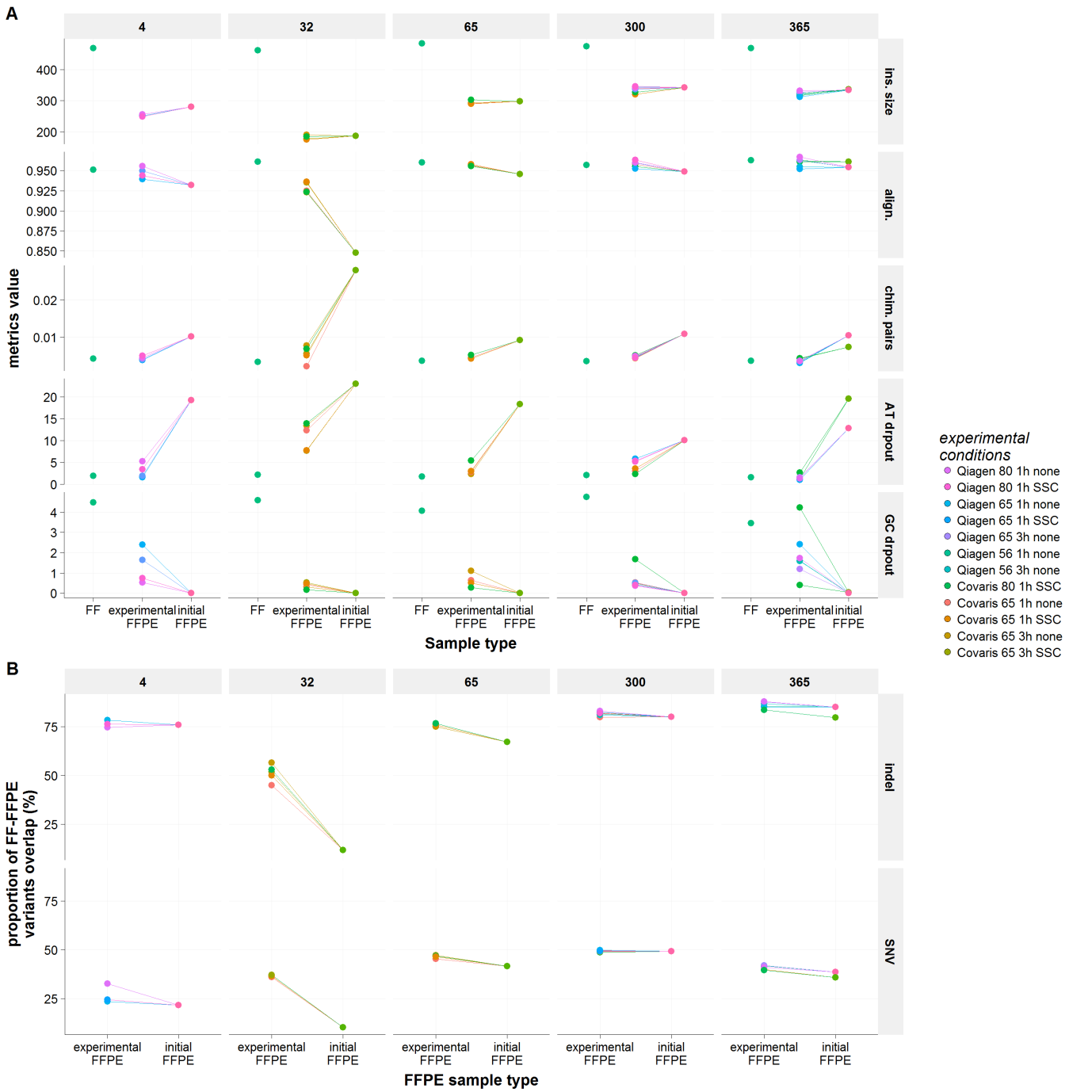


fig. S22 Measurement of the sequencing metrics and variant overlap for experimental FFPE samples.

Comparison of five alignment metrics in five patients between FF samples experimental FFPE and initial FFPE samples (A) and evolution of the proportion of variant overlap (SNV called with Mutect and Strelka and indels by Strelka) between matching initial and experimental FFPE samples (B). Each patient is represented in a column and different DNA extraction conditions are showed by different coloured dots. The overlap between FF and experimental FFPE datasets was higher than in FF and initial FFPE datasets, with the exception of one condition for patient 004 (Qiagen 80°C/1 hour/no buffer addition), where the overlap with FF was 74% for the experimental FFPE dataset and 76% for initial FFPE dataset.

The experimental conditions are described in the following order: DNA extraction kit (Qiagen=QIAamp DNA FFPE Tissue, Covaris=truXTRAC FFPE DNA Kit), reverse crosslinks step temperature and incubation time, addition of buffer (SSC = Saline Sodium Citrate), Initial = FFPE DNA extracted according to the manufacturer’s instructions, experimental = FFPE DNA extracted following a different reverse crosslinking step. Ins. Size = median insert size (bp), align. = Read PF aligned ratio, chim. pairs = Chimeric pairs ratio. AT and GC dropout are expressed in percent.


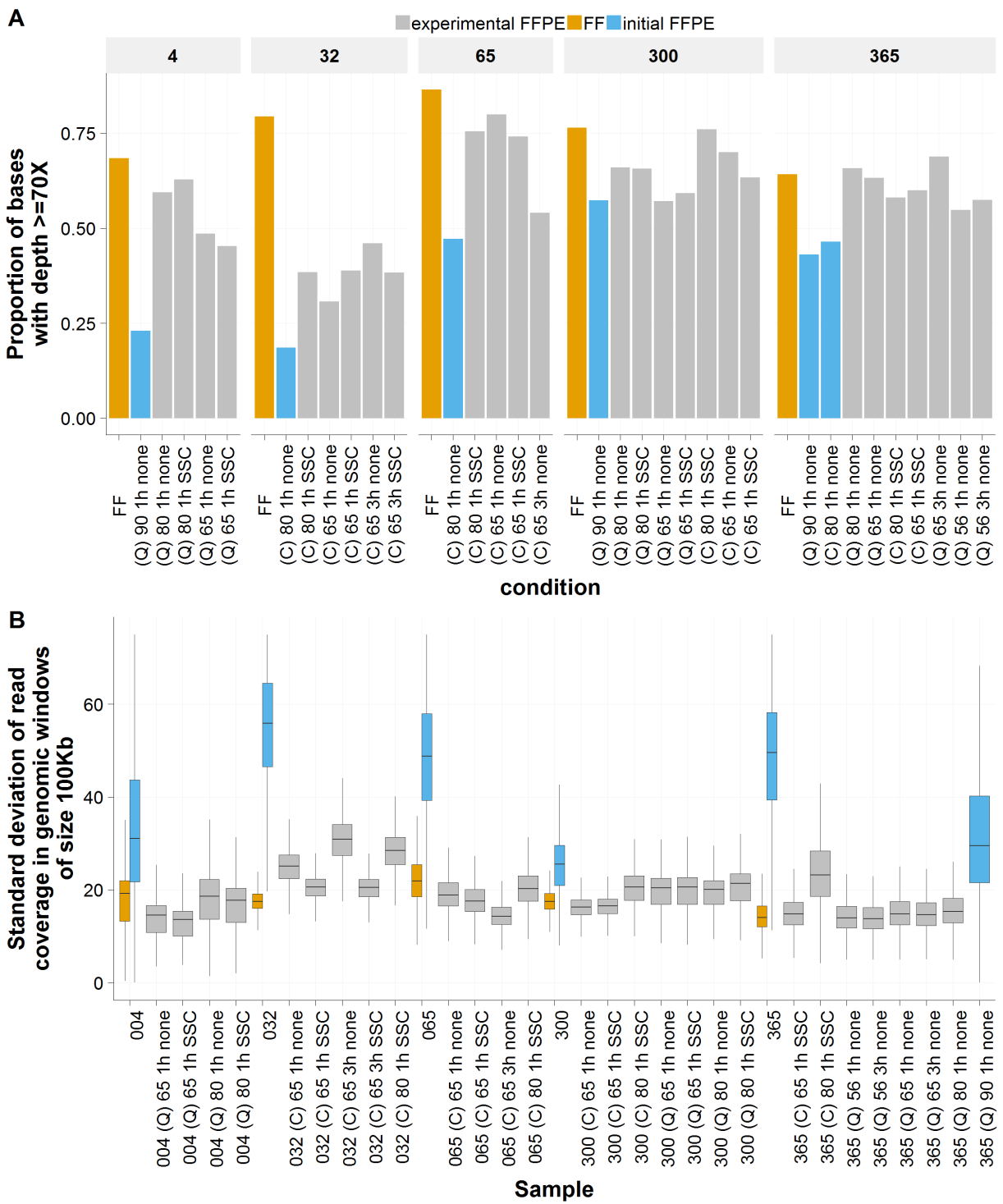


Fig. S 23 FFPE DNA extraction optimisation effect depth of coverage

Proportion of 100 kb windows across the whole genome with a median coverage >= 70 x for FF (A) and distribution of standard deviations of sequencing coverage in 100 kb windows (B) for initial FFPE and experimental FFPE samples for five patients

FF: fresh frozen, FFPE: formalin fixed paraffin embedded


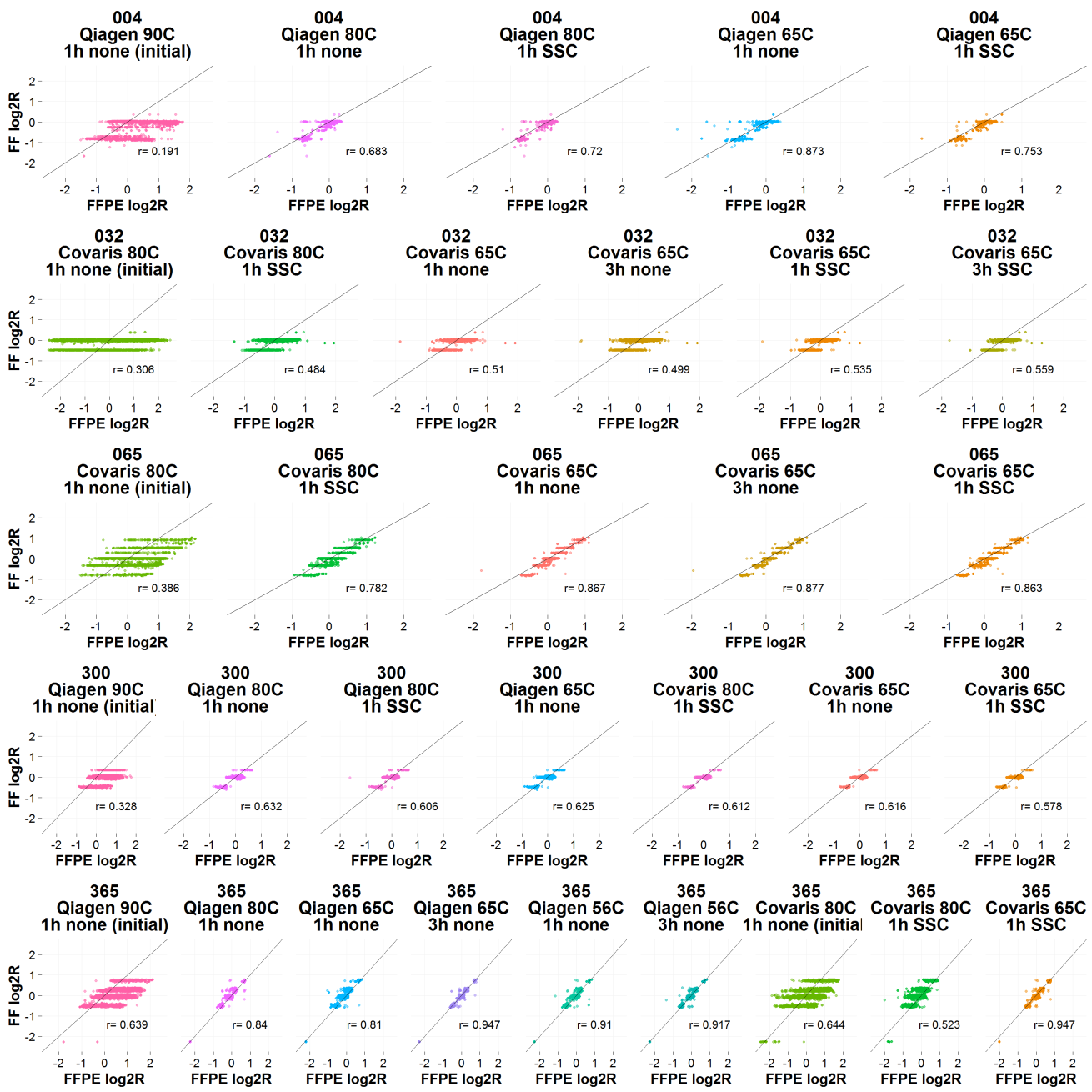


fig. S24 Log_2_R correlation between FF and FFPE samples.

Each row shows the results of one patient. The Spearman correlation coefficient r is indicated in each plot.

FF: fresh frozen, FFPE: formalin fixed paraffin embedded, log2R: log2 ratio


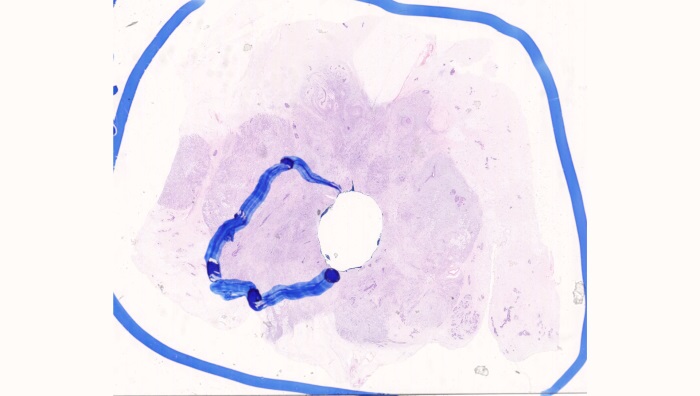


FFPE

FF

fig. S25 H&E section of breast tumour showing where the FF was punched and an adjacent marked area for FFPE extraction

FF: Fresh Frozen sample, FFPE: Formalin-fixed paraffin embedded sample


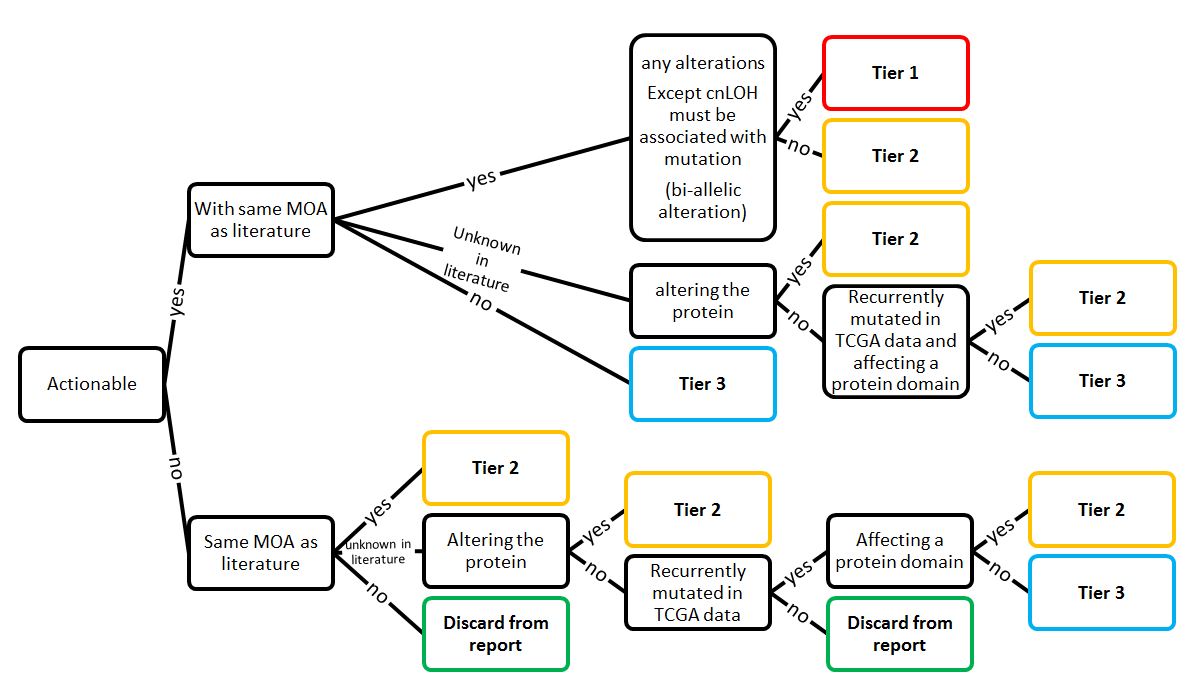


Fig. S 26 System a variant classification for clinical reports

MOA: mechanism of action (gain of function or loss of function of the protein affected from cancer.sanger.ac.uk/cosmic, cbioportal.org and cancer-genetics.org/); Actionable: can influence the patient’s treatment, survival or to gain access to a clinical trial, as described in clinicaltrial.gov or mycancergenome.org; cnLOH: copy neutral loss of heterozygosity, altering the protein: copy number changes, stop codon gains and losses and indels.
